# Homocysteine interferes with Ndufa1 leading to mitochondrial dysfunction through repression of the NAD^+^/Sirt1 pathway in the brain: a possible link between hyperhomocysteinemia and neurodegeneration

**DOI:** 10.1038/s41419-025-07834-3

**Published:** 2025-07-07

**Authors:** Gaoshang Chai, Yuming Mao, Juan Gong, Shuguang Bi, Yuqi Zhang, Jiajun Wu, Liu Yang, Tianlong Gao, Haitian Fu, Chunjing Yu, Caili Ren, Guofu Zhang, Xuming Zhu, Xin Guan, Haoting Yu, Caijing Tang, Yunjuan Nie, Haitao Yu

**Affiliations:** 1 Department of Fundamental Medicine, Wuxi School of Medicine, Jiangnan University, Wuxi, Jiangsu China; 2 MOE Medical Basic Research Innovation Center for Gut Microbiota and Chronic Diseases, Wuxi School of Medicine, Jiangnan University, Wuxi, Jiangsu China; 3 Department of Electrophysiology, Wuhan Children’s Hospital (Wuhan Maternal and Children’s Healthcare Center), Tongji Medical College, Huazhong University of Science and Technology, Wuhan, Hubei China; 4 Nuclear Medicine Center, Jiangnan University Affiliated Hospital, Wuxi, Jiangsu China; 5 Department of Neurology, The Affiliated Mental Health Center of Jiangnan University, Wuxi, Jiangsu China; 6 Department of Clinical Psychiatry, The Affiliated Mental Health Center of Jiangnan University, Wuxi, Jiangsu China; 7 Department of Laboratory Medicine, The Affiliated Wuxi People’s Hospital of Nanjing Medical University, Wuxi, Jiangsu China; 8 Science Center for Future Foods, Jiangnan University, Wuxi, Jiangsu China

**Keywords:** Neurodegeneration, Neurodegeneration

## Abstract

Mitochondrial defects are early pathological changes in neurodegenerative disease (ND). Homocysteine (Hcy) is an independent risk factor for ND. However, whether and how Hcy induces mitochondrial defects during the process of neurodegeneration is unclear. Here, we revealed that Hcy interfered with mitochondrial oxidative phosphorylation (OXPHOS) by inhibiting the mitochondrial electron transport chain (ETC) complex I, resulting in increased levels of reactive oxygen species (ROS) in the hippocampus of rats. Specifically, Hcy suppressed Ndufa1 expression, which is essential for complex I assembly and activation, by interfering with its transcription factor Creb1. Moreover, we found that Hcy induced neurodegeneration-like pathological changes in mitochondria in the brain via the inhibition of the NAD^+^/Sirt1 pathway, including defects in mitochondrial morphology, mitochondrial biogenesis, and mitophagy, ultimately leading to impairments in synapses and cognition, all of which were reversed by Ndufa1 upregulation. Thus, Ndufa1 is a key molecular switch of Hcy-induced mitochondrial damage, and appropriately targeting Ndufa1 or NAD^+^ replenishment may serve as a novel therapeutic strategy for Hcy-induced neurodegeneration and cognitive impairment.

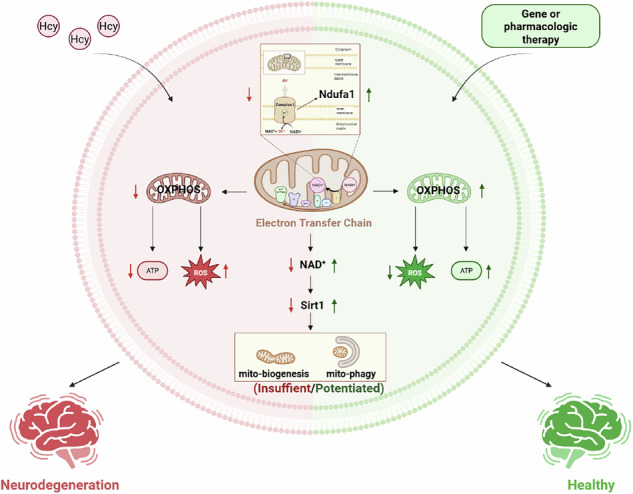

## Introduction

Globally, nearly 50 million people suffer from dementia or cognitive deficits [1]. Given that neurodegeneration is the main factor in cognitive impairment, identifying nongenetic pathogenic events that convert normal aging into neurodegeneration is urgently necessary. Homocysteine (Hcy) is a significant risk factor for neurodegeneration, including Alzheimer’s disease (AD) [2–4]. The serum Hcy level in individuals with dementia is notably increased compared with subjects without any cognitive impairments [5]. Studies have indicated that Hcy can facilitate neuropathological changes in the brain, including cortical thinning, synaptic loss, and deposition of proteins, such as tau, amyloid-β (Aβ), and α-synuclein [6, 7]. Indeed, an abnormal increase in serum Hcy is closely related to cognitive impairment, even before the formation of pathological proteins, indicating other key targets by which Hcy induces neurodegeneration and cognitive impairment [5].

Neurocytes require stable mitochondrial function to generate abundant energy/ATP for their survival, action, and excitatory synaptic transmission. Mitochondrial defects and dysfunction are the hallmark pathological and pathophysiological changes in the brain and contribute to the pathogenesis of neurodegenerative disease (ND) [8]. Emerging findings demonstrate that mitophagy cannot remove impaired mitochondria, leading to the accumulation of dysfunctional mitochondria during neurodegeneration [9]. On the other hand, new mitochondria can also not be produced from existing mitochondria or destroyed mitochondria in the neurodegenerative brain [10]. Thus, both mitophagy and mitochondrial biogenesis are potential targets for the diagnosis of and therapeutic intervention against neurodegeneration and dementia [11, 12].

Mitochondrial biogenesis is regulated by different transcription factors, such as nuclear respiratory factors (NRF1 and NRF2) [13], myocyte enhancer factor 2 (MEF2) [14], and estrogen-related receptors (ERR-α, ERR-β, and ERR-γ) [15]. The regulation of mitochondrial DNA (mtDNA) transcription is facilitated by peroxisome proliferator-activated receptor gamma coactivator 1-alpha (PGC1α) in response to hormones, growth factors, and physical exercise [16]. Activated PGC1α can increase the activity of NRF1 to upregulate mitochondrial biogenesis for metabolic adaptation [17]. Correspondingly, a deficiency of PGC1α/NRF1 is usually associated with mitochondrial biogenesis defects resulting from a decrease in energy metabolism and neurodegeneration [18]. The degradation of mitochondria (mitophagy) is regulated by two major pathways: the PINK1/PARKIN pathway and a set of receptor proteins, including FUNDC1, NIX, and BCL2L13 [19, 20]. Maintaining the homeostasis of mitochondria is of paramount importance for the metabolism and function of nerve cells and the brain [21].

Sirt1 is a nicotinamide adenine dinucleotide (NAD^+^)-dependent deacetylase that can regulate mitochondrial biogenesis and mitophagy [22]. Mitochondrial dysfunction is closely related to neurodegeneration, and the respiratory chain complex I (NADH ubiquinone oxidoreductase) defect is the most important and common enzyme deficiency in mitochondrial diseases related to oxidative phosphorylation (OXPHOS) [23, 24]. The primary function of complex I, which is abundant on the inner mitochondrial membrane, is to facilitate electron transfer to ubiquinone reductase and to oxidize NADH to NAD^+^ [25]. Complex I deficiency is also linked to neurodegenerative disorders such as AD and Parkinson’s disease (PD) [26]. Complex I is composed of 45 subunits, among which Ndufa1 is essential for complex I assembly and activation [27, 28]. Previously, Ndufa1 variants were reported to be associated with neurological diseases [28, 29]. Recently, screening in a large cohort of early-onset dementia (EOD) patients revealed that Ndufa1 variants may play a role in neurodegenerative dementia [30]. Importantly, systematic analysis of differentially expressed genes between AD patients and controls indicated that Ndufa1 could be used as a predictive candidate biomarker for mild cognitive impairment (MCI) and AD [31]. However, the factors that trigger mitochondrial defects in complex I, especially those that target Ndufa1, are poorly understood.

In this study, we identified Hcy-mediated suppression of mitochondrial biogenesis genes, including AMPKα1, PGC1α, NRF1, and TFAM, related to mitochondrial impairment and mitophagy defects in AD. In addition, Hcy impaired energy metabolism by inhibiting mitochondrial OXPHOS, resulting in the upregulation of intracellular reactive oxygen species (ROS) and mitochondrial superoxide (MitoSOX) in the hippocampus. Further exploration of the underlying molecular mechanism revealed that Hcy-induced defects in mitochondrial homeostasis via inhibition of the Sirt1/PGC1α pathway. At the molecular level, Hcy decreased Sirt1 activity by directly inhibiting NAD^+^ metabolism. Hcy interfered with Ndufa1 expression in rat brains and N2a cells. Finally, we demonstrated that Ndufa1 upregulation or NAD^+^ supplementation could reverse the mitochondrial homeostasis defects induced by Hcy. Our findings suggested that Hcy interfered with mitochondrial homeostasis by inhibiting NAD^+^ metabolism via Ndufa1 in the chronic course of neurodegeneration and that NAD^+^ supplementation could ameliorate Hcy-induced neurodegeneration and cognitive impairment.

## Results

### The Hcy-treated hippocampus exhibits transcriptomic changes in the central pathways of mitochondrial function, oxidative stress, and synaptic function

**Fig. 1:**
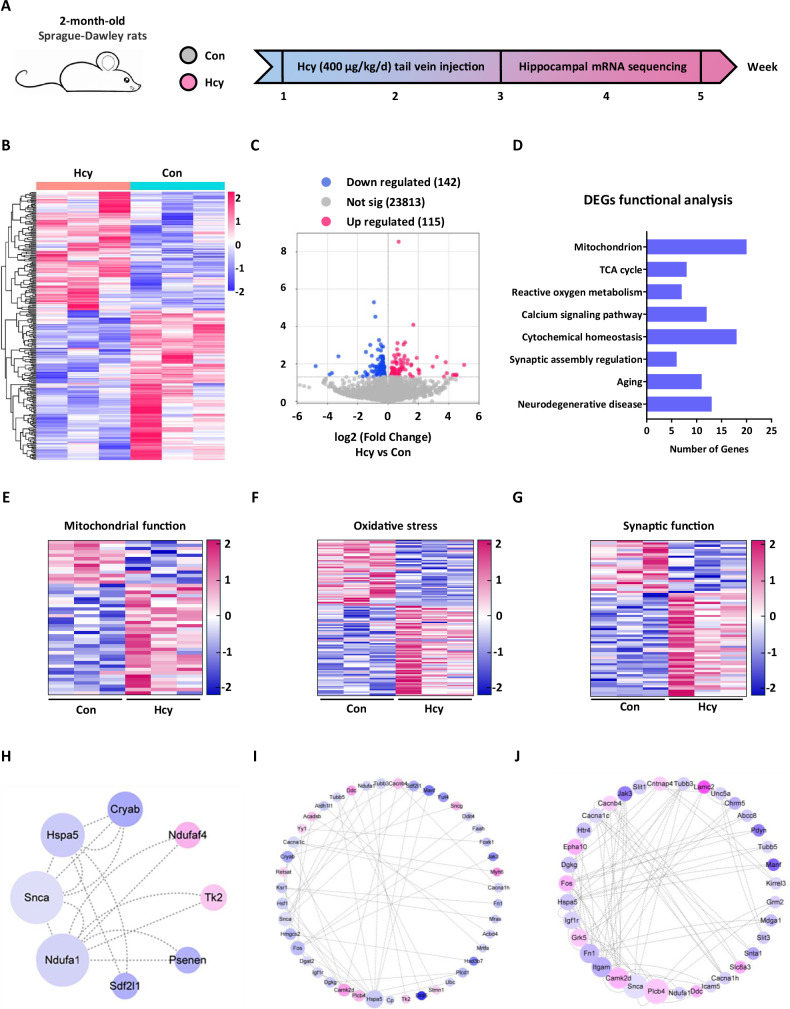
The Hcy-treated hippocampus exhibits transcriptomic changes in the central pathways of mitochondrial function, oxidative stress, and synaptic function. **A** HHcy rat model and hippocampal mRNA sequencing workflow. **B** Heatmap of 257 differently expressed genes (DEGs) in the hippocampus of 2-month-old rats between Con and Hcy groups (*n* = 3 for each group). **C** Volcano plot of mRNA levels changes in 142 downregulated DEGs (blue) and 115 upregulated DEGs (red). **D** The main enrichment terms of DEGs in Con and HHcy rats. **E–G** Heapmaps of the DEGs that were related to mitochondrial function (**E**), oxidative stress (**F**), and synaptic function (**G**). **H–J** Multiple protein interaction analysis showed the key molecule network in different gene sets. The red circle represents upregulated genes and the blue circle represents downregulated genes. The size of the circles was arranged by the value of the betweenness centrality (BC).

Hippocampus is the most commonly affected area in neurodegenerative diseases, including AD, and is closely related to cognitive function [32]. To better understand the genetic status of early symptoms in the hippocampus of hyperhomocysteinemia (HHcy) rats, 2-month-old male Sprague-Dawley (SD) rats were subjected to hippocampal mRNA sequencing after 3 weeks of tail vein injection of PBS or Hcy (400 μg/kg/d) (Fig. 1A) [33]. Differentially expressed genes (DEGs) were identified based on *P*-value < 0.05. A total of 257 DEGs (Fig. 1B) were identified via sequence analysis, 142 of them presented increased expression and 115 presented decreased expression (Fig. 1C). To gain a more comprehensive understanding of the functions of these 257 DEGs, we conducted functional analysis using the DAVID (Database for Annotation, Visualization and Integrated Discovery) bioinformatics database. According to the description of gene function keywords (such as “mitochondrion”, “reactive oxygen metabolism”, “synapses”, and “neurodegeneration”, etc) in DAVID, genes with the same or similar keywords were manually classified into one category (Fig. 1D). Based on the enriched functions from DAVID and pathological characteristics of neurodegenerative diseases, we further classified the related DEGs into three major clusters: mitochondrial function, oxidative stress, and synaptic function (Fig. 1E–G).

Interestingly, the mitochondrial gene set included genes related to OXPHOS (Ndufa1, Ndufaf4, and Cyct), mitochondrial transcription (Mgme1, Mrpl43, Sirt7, and Tk2) and autophagy (Ddit4, Dapk1, and Mras) (Fig. 1E). The above DEGs were also related to oxidative stress (Hmgcs2, Drd5, and Slc4a11) (Fig. 1F) and synaptic function (Ddit4, Drd5, and Nptx1) (Fig. 1G), which may cause ND, such as AD and PD. To visualize the main genes within different gene sets, we constructed protein-protein interaction (PPI) network diagrams and found that one pivotal gene may be the core node in the mitochondrial damage module, which was significantly changed in the Hcy group (Fig. 1H–J). The above data indicated that transcriptomic changes in mitochondrial genes were prominent features in the hippocampus of HHcy rats and that defects in OXPHOS, mitochondrial biogenesis, and mitophagy, as well as a series of mitochondrial dysfunctions induced by Hcy, may specifically be involved.

### Hcy induces mitochondrial dysfunction in the hippocampus of rats

**Fig. 2:**
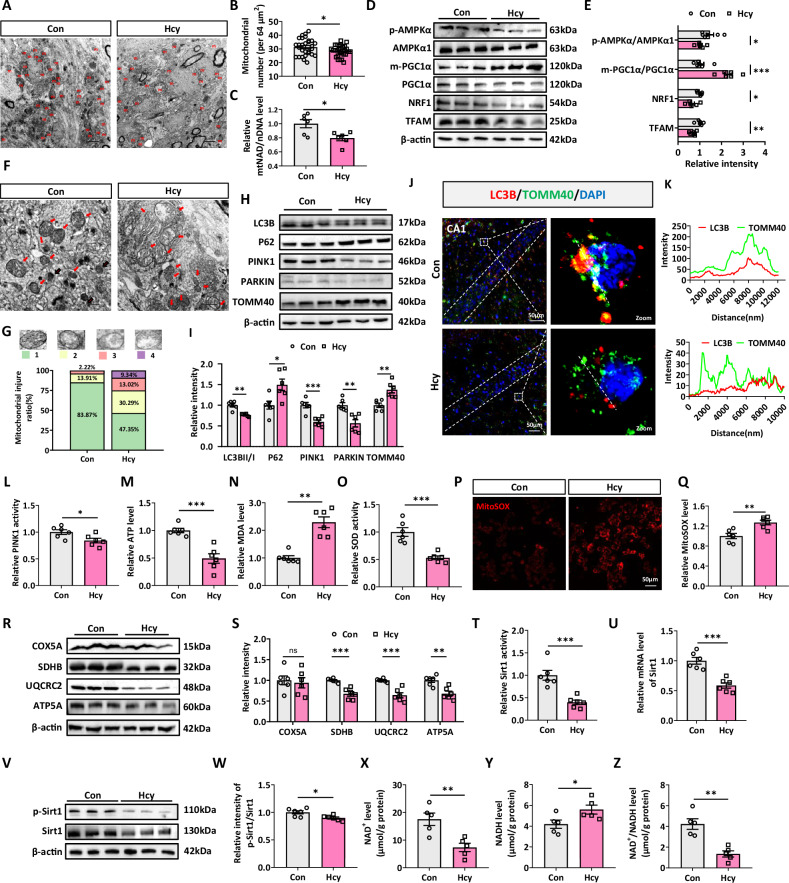
Hcy induces mitochondrial dysfunction in the hippocampus of rats. **A**, **B** Representative TEM images of mitochondria during Hcy treatment. The red “m” in the images represented mitochondria (*n* = 5 for each group, 6 serial sections per rat). **C** Relative mtDNA/nDNA ratio in the hippocampus of Con and HHcy rats. **D**, **E** Western blotting of p-AMPKα1/AMPKα, m-PGC1α/PGC1α, NRF1, and TFAM protein abundance in the hippocampus of Con and HHcy rats. **F**, **G** Red arrows indicated damaged mitochondria. All mitochondria were divided into 4 levels according to the degree of damage from mild to severe. The scoring criteria referred to [74]. **H**, **I** Western blotting measured the expression of LC3B, P62, PINK1, PARKIN, and TOMM40 in the hippocampus of Con and Hcy groups. **J** Representative confocal fluorescence micrographs from the CA1 region of the hippocampus stimulated with or without Hcy and subjected to immunofluorescence labeling of TOMM40 (green) and LC3B (red). **K** The representative line scans were from the LC3B puncta colocalizing with TOMM40. **L** Relative PINK1 activity in the hippocampus of Con and HHcy rats. **M–Q** Relative ATP, MDA, MitoSOX level, and SOD activity in the hippocampus of Con and HHcy rats. **R**, **S** Immunoblot of COX5A, SDHB, UQCRC2, and ATP5A in the hippocampus of Con and HHcy rats. **T** The relative activity of Sirt1 in the hippocampus between Con and Hcy groups was measured using the Sirt1 activity assay. **U** RT-qPCR verification of Sirt1. Gapdh normalized the data. **V**, **W** Immunoblot analysis of p-Sirt1/Sirt1 in the hippocampus of Con and HHcy rats. **X–Z** NAD^+^, NADH, and NAD^+^/NADH levels of the hippocampus in rats between Con and Hcy groups were measured by a related kit (*n* = 5 for each group). Data were presented as mean ± SEM. Unpaired *t*-test was used to analyze the data (*P < 0.05, **P < 0.01, ***P < 0.001; *n* = 6 for each group).

We examined whether the quantity of mitochondria was reduced in the Hcy group. Each rat has 6 serial sections of the hippocampus for mitochondrial counting and 5 rats were chosen for electron transmission microscope (TEM) analysis. Compared with the control group (Con), the result revealed that the number of mitochondria in the rats treated with Hcy was reduced significantly (Fig. 2A, B). To investigate the underlying mechanism of Hcy-induced mitochondrial biogenesis defects, the major regulators of mtDNA/nDNA were determined. We found that the mtDNA/nDNA level was significantly decreased in the Hcy group (Fig. 2C). In addition, decreased p-AMPKα (Thr172) protein (activation form) level and increased m-PGC1α (Lys224) protein (inhibitory form) level in hippocampus suggested that Hcy could inhibit the activities of AMPKα and PGC1α (Fig. 2D, E). Taken together, these data suggested that Hcy-induced defects in mitochondrial biogenesis by effectively repressing key factors that regulated this process.

We also examined the mitochondrial morphology ultrastructure via TEM. Compared with those in Con, the mitochondria in the rat hippocampus were larger and hollower, with empty vacuoles and broken membranes, and even the cristae were disarrayed and sparse (Fig. 2F, G). We investigated whether Hcy damaged the removal of unhealthy mitochondria through mitophagy. The effects of Hcy on mitophagy were examined using confocal microscopy of brain slices. We found that Hcy decreased the colocalized puncta of LC3B and TOMM40 in the hippocampal CA1 region of rats treated with Hcy (Fig. 2J, K). These findings suggested that Hcy inhibited mitophagy in the hippocampus of rats. Compared with those in Con, the levels of mitophagy-associated proteins LC3BII/I in the hippocampus of Hcy-treated rats were significantly lower as determined by western blotting. In contrast, the levels of P62 and TOMM40 increased considerably in the Hcy group (Fig. 2H, I). Furthermore, the levels of PTEN-induced putative kinase 1 (PINK1) and E3 ubiquitin-protein ligase (PARKIN) were also measured. These proteins were involved in mediating mitophagy in ND [34]. Consistently, the protein levels of PINK1 and PARKIN, as well as PINK1 activity, were significantly reduced in rat hippocampal extracts treated with Hcy (Fig. 2H, I, L).

We found that Hcy could induce defective mitochondrial biogenesis and mitophagy. These findings prompted us to explore whether Hcy disturbed energy metabolism in the hippocampus of rats. We initially quantified changes in ATP production within the hippocampus and observed that ATP levels were significantly decreased in the hippocampus of Hcy-treated rats (Fig. 2M), indicating that Hcy could impair energy metabolism. We also tested MDA content, SOD activity and MitoSOX content, the results revealed that Hcy increased the MitoSOX and MDA levels and decreased the SOD activity (Fig. 2N–Q). To investigate the effects of Hcy on the tricarboxylic acid (TCA) cycle, we measured the levels of the mitochondrial ETC proteins COX5A, SDHB, UQCRC2, and ATP5A in the hippocampus. Western blotting revealed that SDHB, UQCRC2, and ATP5A were significantly decreased in the hippocampus of Hcy-treated rats (Fig. 2R, S). To further elucidate the mechanisms by which Hcy inhibits mitochondrial function, we investigated the impact of Hcy on glucose uptake in neural cells. Unexpectedly, the PET/CT results revealed an increase in glucose uptake in the hippocampus after Hcy treatment (Fig. S1A, B). Importantly, increased glucose uptake is consistent with the early imaging phenotype of NDs, such as AD, which may be due to microglial activation, chronic inflammation, and glycolysis [35, 36]. Taken together, these data demonstrated that Hcy impaired energy metabolism (ATP production) by inhibiting mitochondrial OXPHOS in the hippocampus, accompanied by increased MitoSOX and MDA levels, elevated glucose uptake, and other early metabolic pathological characteristics of ND.

Multiple reports have established a crucial role of Sirt1 in mitophagy and mitochondrial biogenesis [37–39]. We investigated whether Hcy-induced mitochondrial dysfunction was associated with Sirt1 activity. Our results indicated that Hcy effectively reduced Sirt1 activity and mRNA levels in the hippocampus of the rats (Fig. 2T, U). In addition, we evaluated Sirt1 activity by monitoring Sirt1 phosphorylation at Ser47 (activation form) and found that Sirt1 activity decreased (Fig. 2V, W). Since Sirt1 is NAD^+^ dependent, we assessed whether Hcy could regulate the level of NAD^+^, the sole key coenzyme of Sirt1. The results showed that Hcy reduced NAD^+^ levels in the hippocampus (Fig. 2X) and the level of NADH was increased in the hippocampus of rats treated with Hcy (Fig. 2Y). The reduction in NAD^+^ was confirmed by assessing the ratio of NAD^+^ to NADH (Fig. 2Z). These findings suggested that Sirt1 is involved in the mechanism underlying Hcy-induced mitochondrial dysfunction via the inhibition of NAD^+^ synthesis.

### Ndufa1 mediates Hcy-induced mitochondrial dysfunction in the brain

**Fig. 3:**
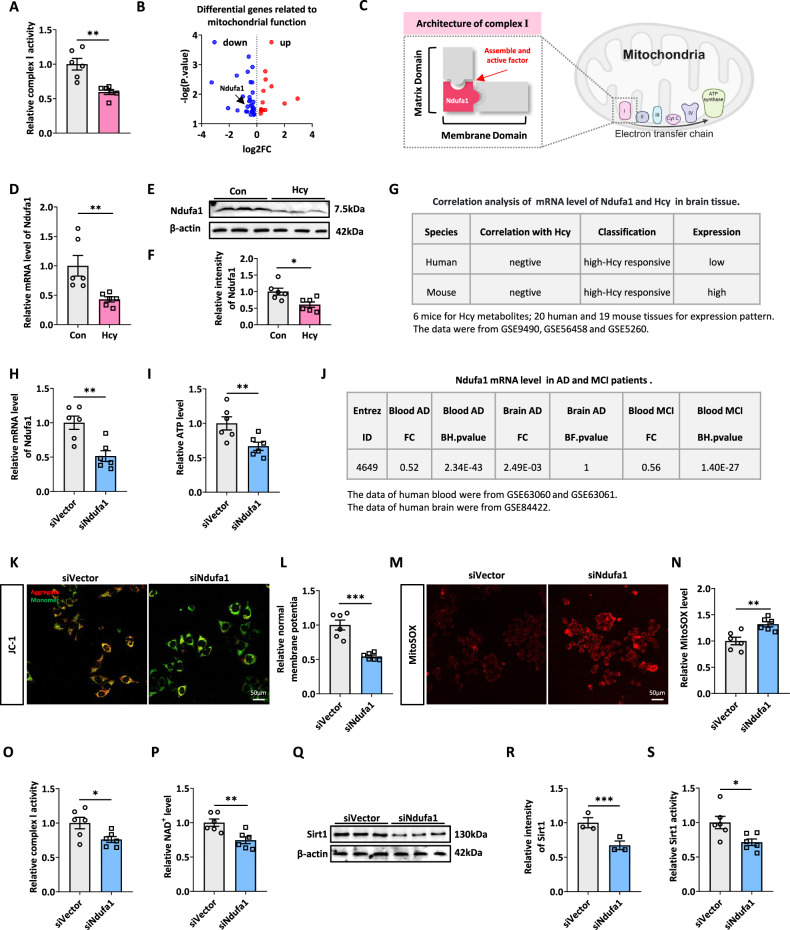
Ndufa1 mediates Hcy-induced mitochondrial dysfunction in the brain. **A** Relative complex I activity of hippocampus in rats between Con and Hcy groups. **B** Volcano plot of mitochondrial function-related DEGs. The DEGs in the volcano plot were derived from the mitochondrial gene dataset in Fig. 1D. Blue indicated downregulated genes and red indicated upregulated genes. The location of the arrow indicated Ndufa1. **C** Diagram of the role of Ndufa1 as an assembly and active factor in complex I of the mitochondrial ETC. **D** The mRNA expression of Ndufa1 in the hippocampus of Con and HHcy rats. The data were normalized by Gapdh. **E**, **F** Images and quantification of immunoblotted Ndufa1 signal in the hippocampus from Con and HHcy rats. **G** The mRNA level of Ndufa1 was negatively correlated with Hcy content in humans and mice, and the brain was defined as “high-Hcy responsive” tissue. The conclusion was from [40]. **H** The mRNA level of Ndufa1 in N2a cells decreased significantly after transfection with siNdufa1. **I** The relative ATP level was decreased in N2a cells treated with siNdufa1. **J** The expression level of Ndufa1 in brain tissue and blood from AD and MCI patients showed a statistically significant and decreasing trend. The conclusion was from [31]. **K–N** Following siNdufa1 treatment, both mitochondrial membrane potential (MMP) and MitoSOX levels were elevated in N2a cells. **O**, **P** Complex I activity and NAD^+^ level were detected by relevant kits. **Q–S** Western blotting and an ELISA kit were used to detect Sirt1 expression and activity. Data were presented as mean ± SEM. Unpaired *t*-test was used to analyze the data (*P < 0.05, **P < 0.01, ***P < 0.001; *n* = 6 for each group).

To clarify the molecular mechanism by which Hcy inhibited NAD^+^ synthesis, we further determined the effect of Hcy on mitochondrial ETC complex I (NADH ubiquinone oxidoreductase). We found that Hcy treatment decreased the activity of complex I (Fig. 3A). To explore the mechanisms underlying the reduced activity of complex I induced by Hcy, we analyzed the hippocampal mRNA sequencing data again to identify the key regulatory molecules. Sequence analysis revealed that Ndufa1, which is essential for complex assembly and activation, was significantly reduced in the hippocampus of Hcy-treated rats (Fig. 3B). Ndufa1 played an important role in the assembly and activity of complex I (Fig. 3C). RT-qPCR and western blotting verified that Hcy suppressed Ndufa1 expression in the hippocampus of the rats (Fig. 3D–F). These data indicated that Hcy inhibited complex I activity by suppressing Ndufa1.

A novel hypothesis establishment of Hcy-suppressive mitochondrial ETC complex genes and tissue expression profile revealed that there was a negative linear correlation between Ndufa1 mRNA and Hcy levels in human and mouse brains (Fig. 3G). Meanwhile, the study summarizes numerous studies involving Ndufa1 mutations with inhibitory effects on complex I activity and assembly function in disease and experimental models, including ND [40]. From another systematic analysis and biomarker study for AD, researchers found that Ndufa1 was significantly reduced in blood and brain of AD and MCI (Fig. 3J) [41].

To further verify the importance of Ndufa1 for mitochondrial function, we used siRNA to establish Ndufa1 knockdown N2a cell line. RT-qPCR results of mRNA levels in transfected cells showed that the siNdufa1 could effectively knock down the mRNA level of Ndufa1 (Fig. 3H). At the same time, lower Ndufa1 resulted in the appearance of a series of indicators of mitochondrial damage, such as the decrease of ATP and mitochondrial membrane potential (MMP), and the increase of MitoSOX (Fig. 3I, K–N). Meanwhile, the activity of complex I and NAD^+^ level was significantly reduced (Fig. 3O, P), and further led to Sirt1 expression and activity downregulated (Fig. 3Q–S). The above data suggested that Ndufa1 mediates Hcy-induced mitochondrial dysfunction in the brain.

### Upregulating Ndufa1 rescues Hcy-induced cognitive impairment in rats

**Fig. 4:**
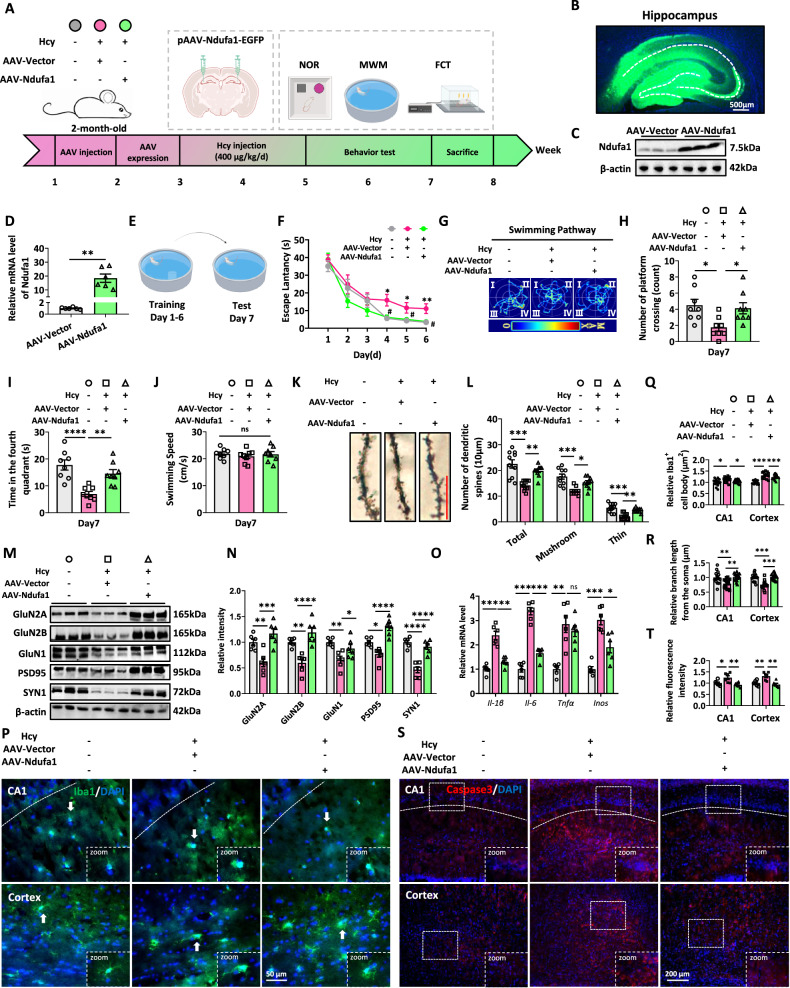
Upregulating Ndufa1 rescues Hcy-induced cognitive impairment in rats. **A** The 24 2-month-old male SD rats were randomly divided into 3 groups and the workflow demonstrated the experimental processes. **B** The rats received bilateral hippocampal injections of AAV-virus in the hippocampal CA3 region. The virus expression (green fluorescence) in the hippocampus was observed by a fluorescence microscope. **C**, **D** The mRNA or protein levels of Ndufa1 were detected and quantified by RT-qPCR and western blotting. **E** MWM showed that HHcy rats had poor spatial and learning memory ability. **F** Escape latency to the hidden platform between days 1–6 (The platform is in the 4th quadrant.). **G** The swimming pathway was used to locate the platform on day 7. **H** Number of crossings in the 4th quadrant on day 7. **I** Time in the 4th quadrant on day 7. **J** Swimming speed of rats on day 7. **K**, **L** Typical dendrites of tertiary branch hippocampal pyramidal neurons with Golgi staining and quantitative examination of the overall density of dendritic spines and the density of thin-shaped and mushroom-shaped spines in the hippocampal CA1 region (*n* = 5 for each group, 2 neurons per rat). **M**, **N** Expression of GluN2A, GluN2B, GluN1, PSD95, and SYN1 was measured by western blotting (*n* = 6 for each group). **O** Relative mRNA level of Il-1β, Il-6, Tnfα, and Inos in the hippocampus of rats (*n* = 6 for each group). **P**, **Q**, **S** IBA1 staining in the CA1 region of the hippocampus and cortex from rats. Microglial activation was assessed based on the soma size and branch length of IBA1^+^ cells in the hippocampus and cortex from rats (*n* = 3 for each group, 5 cells from each rat). **R**, **T** Cleaved Caspase3 staining was used to label the CA1 region of the hippocampus and cortex in rats (*n* = 6 for each group). Data were presented as mean ± SEM. Unpaired *t*-test for D. Two-way repeated measures ANOVA followed by Bonferroni’s post hoc test for (**F**). One-way ANOVA followed by Bonferroni’s post hoc test for others (*P < 0.05, **P < 0.01, ***P < 0.001, ****P < 0.0001, ns no significance; *n* = 8 for each group).

Our previous research revealed that Hcy impaired the spatial memory of rats [42]. Based on Ndufa1 is related to mitochondrial function and the importance of mitochondrial function for nerve cells, we investigated the effects of the upregulation of Ndufa1 on cognitive impairment in Hcy-treated rats. To further verify the effects of Ndufa1 on Hcy-induced cognitive impairment in rats, we constructed an adeno-associated virus (AAV-Vector and AAV-Ndufa1) and injected into the hippocampus of the rats before Hcy tail injection (Fig. 4A). Three weeks after AAV injection, robust virus transfection was observed via enhanced green fluorescent protein (EGFP) expression (Fig. 4B), and the expression efficiency was confirmed by the significantly upregulated protein and mRNA levels of Ndufa1 in the hippocampus of rats, as measured by western blotting (Fig. 4C) and RT-qPCR (Fig. 4D).

We first determined the influence of Ndufa1 upregulation on spatial memory via the Morris water maze (MWM) test (Fig. 4E). The Hcy+AAV-Vector rats exhibited a significantly longer escape latency than the control rats did on the 4th–6th days (Fig. 4F) and presented fewer platform crossings and spent less time in the platform quadrant (Fig. 4G–I) on day 7. However, there was no difference in the rats’ swimming speed (Fig. 4J). The impact of Hcy on short-term memory was evaluated using the novel object recognition test (NOR) (Fig. S2A). The rats were allowed to explore 2 objects that were identical in an open field box during the training period. The exploration time during the familiar period did not significantly differ among the groups (Fig. S2B). During the test period, another object that was new to the rats replaced one of the old familiar objects, and the recognition time decreased significantly in the Hcy+AAV-Vector rats compared with that in Con (Fig. S2B). However, the upregulation of Ndufa1 reversed the changes in the recognition time (Fig. S2B). Intergroup comparisons revealed that Hcy+AAV-Vector rats were less curious toward novel objects than control and Hcy+AAV-Ndufa1 groups (Fig. S2C). Representative findings from the NOR test consistently demonstrated a preference for exploring novel objects, as indicated by the discrimination index (Fig. S2D). This suggested a strong inclination toward novelty exploration in experimental subjects. Con and Hcy+AAV-Ndufa1-treated HHcy rats showed stronger attention and attentional bias toward novel objects, whereas Hcy+AAV-Vector rats were less interested. We also analyzed behavior performance using a fear conditioning test (FCT) for associative memory involving the hippocampus (Fig. S2E). Compared with control, Hcy+AAV-Vector rats presented significantly shorter freezing responses in recent (2 h) and remote (24 h) retrieval tests, suggesting impaired memory, which was rescued by the AAV-Ndufa1 (Fig. S2F–H). These results suggested that upregulating Ndufa1 could ameliorate Hcy-induced cognitive deficits.

The degeneration of dendritic spines is an important hallmark of neurodegeneration. A remarkable reduction in the density of dendritic spines, including mushroom-shaped spines and thin spines, in the hippocampal CA1 region was observed in the Hcy treatment group, and upregulation of Ndufa1 ameliorated the decrease in the density of dendritic spines (Fig. 4K, L). Western blotting revealed that upregulation of Ndufa1 dramatically attenuated Hcy-induced synapse-associated protein reduction, including N-methyl-D-aspartic acid receptor type 2 A (GluN2A), N-methyl-D-aspartic acid receptor type 2B (GluN2B), glutamate receptor ionotropic (GluN1), postsynaptic density protein 95 (PSD95), and Synapsin 1 (SYN1) (Fig. 4M, N). To explore whether Hcy could cause neuroinflammation, RT-qPCR was used to quantify pro-inflammatory factors (Il-1β, Il-6, Tnfα, Inos) in the hippocampus of rats. The results showed that the levels of pro-inflammatory factors in the Hcy+AAV-Vector group were significantly higher than that in the Hcy+AAV-Ndufa1 group, suggesting the existence of neuroinflammation in Hcy+AAV-Vector rats (Fig. 4O). Neuroinflammation caused by activation of microglia is one of the reasons of nerve cell apoptosis [43]. Therefore, we further explored the effect of Hcy on microglia. IBA1 staining showed that Hcy could enlarge the cell body and reduce branches’ length of microglia (Fig. 4P–R). Meanwhile, we stained the hippocampus and cortex by activated Caspase3 (p17/p19) to observe cell death. The results showed that Hcy caused apoptosis in both the CA1 region of hippocampus and cortex (Fig. 4S, T). In general, these findings suggested that the upregulation of Ndufa1 effectively mitigated Hcy-induced cognitive impairment in the hippocampus of rats.

### Upregulating Ndufa1 rescues Hcy-induced mitochondrial dysfunction

**Fig. 5:**
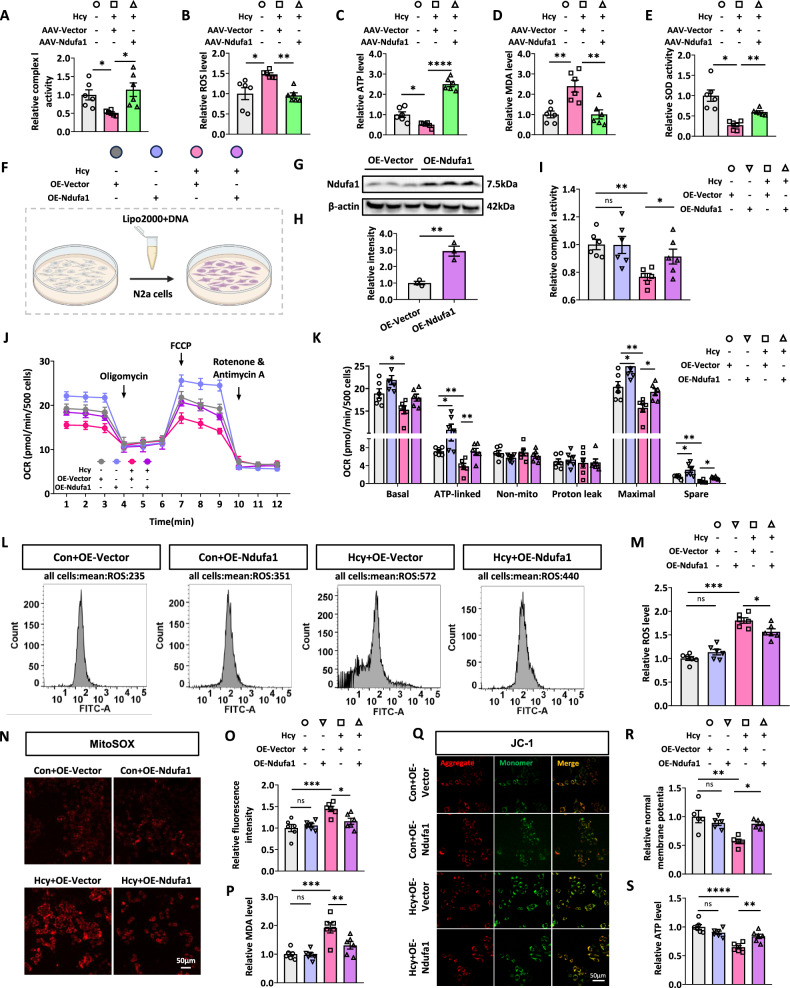
Upregulating Ndufa1 rescues Hcy-induced mitochondrial dysfunction. **A–E** The levels or activities of complex I, ATP, ROS, MDA, and SOD in the hippocampus of rats were measured using the corresponding kits. **F** The procedure of plasmid (Vector-pcDNA3.1 or Ndufa1-pcDNA3.1) transfection was carried out according to the instructions of the Lipo2000 agent. **G**, **H** The protein levels of Ndufa1 were detected and quantified by western blotting (*n* = 3 for each group). **I** The related kits tested relatively complex I activity after overexpressing Ndufa1 or Vector in N2a cells. **J** Respiratory oxygen consumption rate (OCR, pmol/min/500 cells) determined by Seahorse real-time cell metabolic analysis in N2a cells treated with/without Hcy and with/without OE-Ndufa1 plasmid. The cells were stimulated with 1.5 μM oligomycin (Oligo), 2 μM FCCP, and 0.5 μM antimycin A (Ant A) every 20 min during the test. **K** Measurement of basal respiration, ATP-linked respiration, non-mitochondrial respiration, proton leakage, maximum respiration, and spare respiration was analyzed by the software Wave Pro (Agilent, USA). **L**, **M** Representative flow cytometric analysis of mean content of ROS targeted by FITC-A from N2a cells. The data were analyzed by FlowJo (v7.5, USA) (*n* = 5 for each group). **N**, **O** MitoSOX was used to detect mitochondrial superoxide in N2a cells. **Q**, **R** MMP in N2a cells was measured by JC-1 dye. Red fluorescence (Aggregate) indicated higher MMP and green fluorescence (Monomer) indicated lower MMP (*n* = 5 for each group). **P**, **S** The related kits tested relative ATP and MDA levels after overexpressing Ndufa1 or Vector in N2a cells. Data were presented as mean ± SEM. One-way ANOVA followed by Bonferroni’s post hoc was used to analyze the data (*P < 0.05, **P < 0.01, ***P < 0.001, ***P < 0.0001, ns no significance; *n* = 6 for each group).

To further verify the effects of Ndufa1 on Hcy-induced mitochondrial dysfunction, we measured the levels or activities of complex I, ROS, ATP, MDA, and SOD. With Ndufa1 upregulation, the reduced activity of complex I was effectively reversed in the hippocampus of Hcy+AAV-Vector rats (Fig. 5A). Upregulation of Ndufa1 ameliorated the Hcy-induced increase in intracellular ROS, MDA production, decreased ATP levels and SOD activity (Fig. 5B–E).

To investigate the role of Ndufa1 in Hcy-induced mitochondrial defects, we established N2a cell lines with transient overexpression of Ndufa1 (Ndufa1-pcDNA3.1) or the vector (control). We measured the expression levels of Ndufa1 and complex I activity changes. The Ndufa1 expression and complex I activity were significantly elevated in N2a cells with transient overexpression of Ndufa1 (Fig. 5F–H). In N2a cells, increasing Ndufa1 ameliorated the Hcy-induced reduction in complex I activity (Fig. 5I). To investigate the effects of Ndufa1 upregulation on energy metabolism in Hcy-treated N2a cells, a Seahorse extracellular flux assay was performed. We found that upregulation of Ndufa1 effectively improved the oxygen consumption rate (OCR), as shown by increased ATP-linked respiration, maximum respiration, and spare respiration in Hcy-treated N2a cells (Fig. 5J, K). Meanwhile, Ndufa1 overexpression also increased ATP-linked respiration, maximum respiration, and spare respiration in the absence of Hcy (Fig. 5J, K). Consistently, Ndufa1 upregulation increased ATP levels and the mitochondrial membrane potential (MMP) and decreased intracellular ROS, MitoSOX, and MDA production in Hcy-treated N2a cells (Fig. 5L–S). However, Ndufa1 had no significant effect on these indicators of mitochondrial function without Hcy treatment (Fig. 5L–S). These data suggested that upregulating Ndufa1 rescued Hcy-induced mitochondrial dysfunction.

### Upregulating Ndufa1 rescues Hcy-induced mitochondrial biogenesis and mitophagy defects

**Fig. 6:**
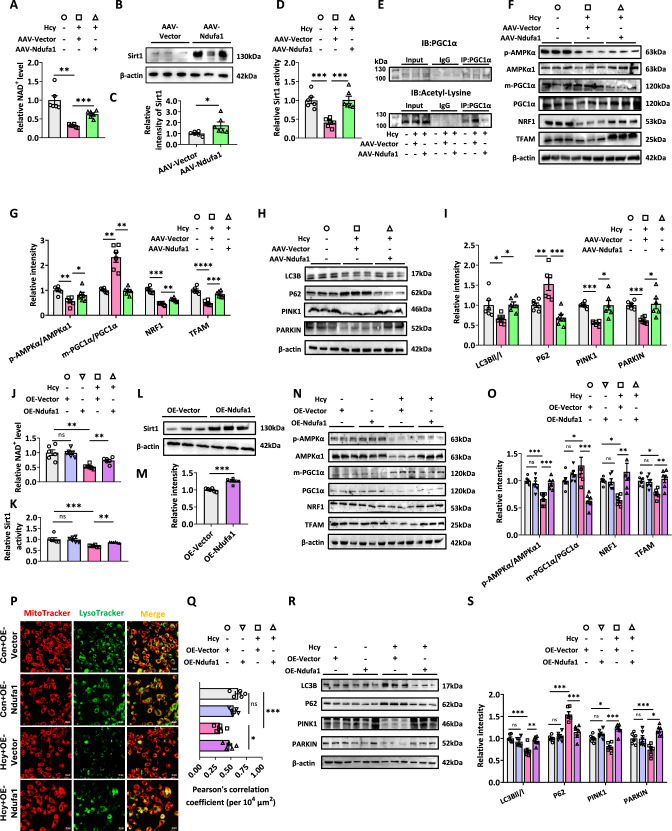
Upregulating Ndufa1 rescues Hcy-induced mitochondrial biogenesis and mitophagy defects. **A–C** The NAD^+^ and Sirt1 levels were detected and quantified by RT-qPCR, western blotting, and a related kit. **D** Meanwhile, Sirt1 activity was measured using an ELISA kit for Sirt1. **E** PGC1α acetylation levels in the hippocampus of rats in 3 groups. First, PGC1α immunoprecipitation (IP) was performed. Total PGC1α and acetylated PGC1α were then detected by western blotting (IB) using anti-PGC1α and anti-acetyl-lysine antibodies, respectively (*n* = 3 for each group). **F**, **G** Western blotting measured the expression of p-AMPKα/AMPKα1, m-PGC1α/PGC1α, NRF1, and TFAM in the hippocampus of rats. **H**, **I** In parallel, western blotting quantified mitophagy-related protein content in the hippocampus. β-actin was used as an internal reference protein for correction. **J** The related kits tested relative NAD^+^ levels after transfecting in N2a cells. **K–M** The protein and activity levels of Sirt1 were detected and quantified by western blotting and an ELISA kit. **N**, **O** SDS-PAGE and IB analysis of p-AMPKα1/AMPKα, m-PGC1α/PGC1α, NRF1, and TFAM in N2a cells. **P**, **Q** Representative confocal live images of mito-tracker (red) and lyso-tracker (green) targeting mitochondria in N2a cells. Cells were kept untreated or treated with Hcy and/or plasmid. Qualification of the Pearson’s correlation coefficient was calculated by Image J (*n* = 5 for each group). **R**, **S** SDS-PAGE and IB analysis of LC3B, P62, PINK1, and PARKIN in N2a cells. Data were presented as mean ± SEM. Unpaired *t*-test for (**C** and **M**). One-way ANOVA followed by Bonferroni’s post hoc was used for others (*P < 0.05, **P < 0.01, ***P < 0.001, ****P < 0.0001, ns no significance; *n* = 6 for each group).

We subsequently investigated the mechanisms of Hcy-induced mitochondrial dysfunction and whether the upregulation of Ndufa1 could ameliorate these changes. With the upregulation of Ndufa1, the decreased levels of NAD^+^ and Sirt1 protein and activity induced by Hcy were significantly reversed in the rat hippocampus (Fig. 6A–D). Moreover, the Hcy-induced inhibition of PGC1α deacetylation in the hippocampus of rats was reversed by the upregulation of Ndufa1 (Fig. 6E). We next examined the protein levels of p-AMPKα/AMPKα1, m-PGC1α/PGC1α, NRF1, and TFAM. We found that upregulating Ndufa1 reversed the decreased changes induced by Hcy (Fig. 6F, G). In addition, the mtDNA/nDNA level increased in Hcy+AAV-Ndufa1 group compared to Hcy+AAV-Vector group (Fig. S3A). These data suggested that upregulating Ndufa1 rescued Hcy-induced mitochondrial biogenesis defects in rats. Moreover, we also observed that the upregulation of Ndufa1 attenuated Hcy-induced mitophagy defects. Western blotting revealed that the levels of LC3BII/I, P62, PINK1, and PARKIN were increased in the hippocampus of Hcy-treated rats, whereas the levels of P62 were significantly decreased (Fig. 6H, I). Furthermore, overexpression of Ndufa1 rescued the inhibition of PINK1 activity by Hcy (Fig. S3C). Together, these data indicated that upregulating Ndufa1 rescued Hcy-mediated disruption of mitochondrial homeostasis in rats.

With increasing complex I activity, Ndufa1 upregulation significantly increased NAD^+^ and Sirt1 levels and activity in Hcy-treated N2a cells (Fig. 6J–M). To further explore the effect of Ndufa1 on N2a cells, empty vectors or plasmids overexpressing Ndufa1 were transfected. After 48 h, the cells were treated with PBS (in Con group) and 100 μM Hcy (in Hcy group) for 24 h. Upregulation of Ndufa1 reversed the Hcy-induced decreases in the protein levels of p-AMPKα/AMPKα1, m-PGC1α/PGC1α, NRF1, and TFAM (Fig. 6N, O). Meanwhile, the mtDNA/nDNA level was increased in Hcy+OE-Ndufa1 group compared to Hcy+OE-Vector group (Fig. S3B). However, overexpression of Ndufa1 did not significantly upregulate the expression of mito-biogenesis related genes without Hcy treatment (Fig. 6N, O). These data suggested that upregulating Ndufa1 rescued Hcy-induced mitochondrial biogenesis defects.

We further analyzed the changes in mitophagy flux by using Lyso-Tracker to green-label (100 nM) autophagosomes and Mito-Tracker to red-label (100 nM) mitochondria. Confocal microscopy revealed that the colocalization of Mito-Tracker (red) with Lyso-Tracker (green) was increased in Hcy-treated N2a cells with transient upregulation of Ndufa1 (Fig. 6P, Q). However, overexpression of Ndufa1 had no significant effect on mitophagy without Hcy (Fig. 6P, Q). These results suggested that the upregulation of Ndufa1 ameliorated Hcy-induced mitophagy defects. Subsequent western blotting revealed that LC3BII/I levels were increased in Hcy-treated N2a cells with transient upregulation of Ndufa1, whereas P62 levels were significantly decreased (Fig. 6R, S). We also measured changes in the levels of PINK1/PARKIN pathway components, which are involved in mitophagy. We observed that the upregulation of Ndufa1 increased PINK1 and PARKIN expression compared to the levels in Hcy+OE-Vector N2a cells (Fig. 6R, S). Meanwhile, overexpression of Ndufa1 rescued the inhibition of PINK1 activity by Hcy in N2a cells. However, overexpression of Ndufa1 without Hcy treatment did not affect the activity of PINK1 (Fig. S3D). These data suggested that upregulating Ndufa1 rescued Hcy-induced mitochondrial biogenesis and mitophagy defects.

### NAD^+^ supplementation attenuates Hcy-induced cognitive impairment and mitochondrial impairment in rats

**Fig. 7:**
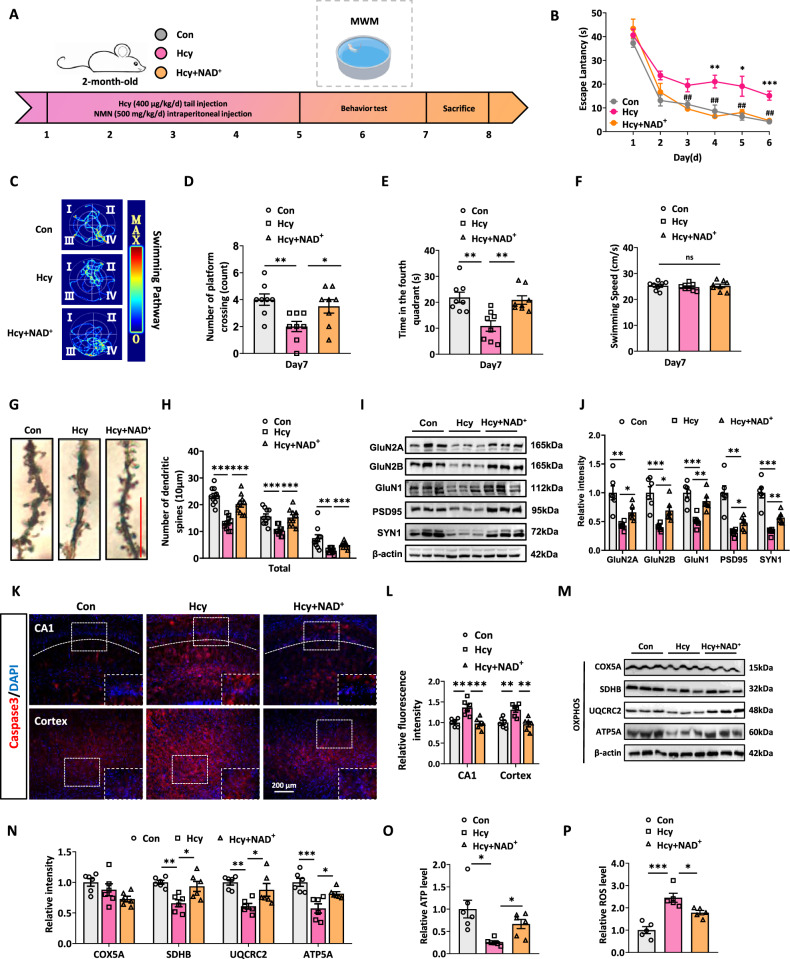
NAD^+^ supplementation attenuates Hcy-induced cognitive inpairment and mitochondrial dysfunction in rats. **A** The 24 2-month-old male SD rats were randomly divided into 3 groups as Con, Hcy, and Hcy+NAD^+^. **B–F** MWM showed that Hcy+NAD^+^ rats had better spatial memory and learning ability than HHcy rats. **G**, **H** Typical dendrites of tertiary branch hippocampal pyramidal neurons with Golgi staining and quantitative examination of the overall density of dendritic spines and the density of thin-shaped and mushroom-shaped spines in the hippocampus CA1 region (*n* = 5 for each group, 2 neurons per rat). **I**, **J** Expression of GluN2A, GluN2B, GluN1, PSD95, and SYN1 was measured by western blotting (*n* = 6 for each group). **K**, **L** Cleaved Caspase3 was used to stain the CA1 region of the hippocampus and cortex in rats (*n* = 6 for each group). **M**, **N** Expression of COX5A, SDHB, UQCRC2, and ATP5A in the hippocampus of Con, Hcy, and Hcy+NAD^+^ rats was measured by western blotting (*n* = 6 for each group). **O**, **P** The relative ATP and ROS levels in the hippocampus were measured using the corresponding kits (*n* = 6 for each group). Data were presented as mean ± SEM. Two-way repeated measures ANOVA followed by Bonferroni’s post hoc test for (**B**). One-way ANOVA followed by Bonferroni’s post hoc was used for others (*P < 0.05, **P < 0.01, ***P < 0.001, ns no significance; *n* = 8 for each group).

Hcy decreased Sirt1 activity by directly inhibiting NAD^+^ synthesis, and the upregulation of Ndufa1 attenuated Hcy-induced defects in mitochondrial homeostasis by improving NAD^+^ synthesis. We investigated whether nicotinamide adenine nucleotide (NMN) supplementation through intraperitoneal injection (500 mg/kg/d) for 3 weeks could attenuate Hcy-induced neurodegeneration and mitochondrial impairment (Fig. 7A). With NAD^+^ supplementation, improved learning and memory abilities were shown by reduced escape latency during the training trials, increased crossing numbers, and increased time spent in the target quadrant (Fig. 7B–F). Supplementation with NAD^+^ rescued the number of dendritic spines, including mushroom-shaped and thin spines (Fig. 7G, H). Western blotting revealed that NAD^+^ supplementation dramatically attenuated the reduction in the levels of Hcy-induced synapse-associated proteins, including GluN2A, GluN2B, GluN1, PSD95, and SYN1 (Fig. 7I, J). To further investigate the impact of NAD^+^ on cell survival, Caspase3 staining was performed on the hippocampus and cortex to assess cell death. The Caspase3 (p17/p19) staining showed that NAD^+^ supplementation alleviated Hcy-induced apoptosis in the CA1 region of hippocampus and cortex (Fig. 7K, L). In general, these results suggested that NAD^+^ supplementation rescued Hcy-induced neurodegeneration in the hippocampus of rats.

We then investigated whether NAD^+^ supplementation would normalize Hcy-impaired mitochondrial function. In Hcy groups, the downregulation of the protein levels of OXPHOS (SDHB, UQCRC2, ATP5A) induced by Hcy was reversed after increasing the NAD^+^ level, with increased ATP levels and reduced levels of ROS (Fig. 7M–P). These data strongly indicated that NAD^+^ supplementation normalized Hcy-induced mitochondrial defects in rats. The reduced level of Sirt1 in the hippocampus of Hcy-treated rats was effectively reversed by NAD^+^ supplementation (Fig. S4A–D). We thus investigated the roles of NAD^+^ supplementation in Hcy-induced mitochondrial biogenesis and mitophagy defects. There were 5 animals, with 6 slices obtained from each animal for TEM analysis. The result revealed that rats treated with NAD^+^ presented a significant increase in the number of mitochondria (Fig. S4E, F). In addition, the mtDNA/nDNA level increased significantly in Hcy+NAD^+^ group compared to Hcy group (Fig. S4G). Western blotting revealed that the protein levels of AMPKα1, PGC1α, NRF1, and TFAM were also reversed in NAD^+^-supplemented HHcy rats (Fig. S4H, I). We then investigated whether NAD^+^ supplementation would normalize Hcy-impaired mitophagy. We found that supplementation with NAD^+^ reversed the changes in the protein levels of LC3BII/I, P62, PINK1, PARKIN, and TOMM40 induced by Hcy (Fig. S4J, K). These data indicated that NAD^+^ supplementation attenuated Hcy-induced cognitive impairment and mitochondrial dysfunction in rats.

### Hcy decreases the expression level of Ndufa1 by reducing the expression of the transcription factor Creb1

**Fig. 8:**
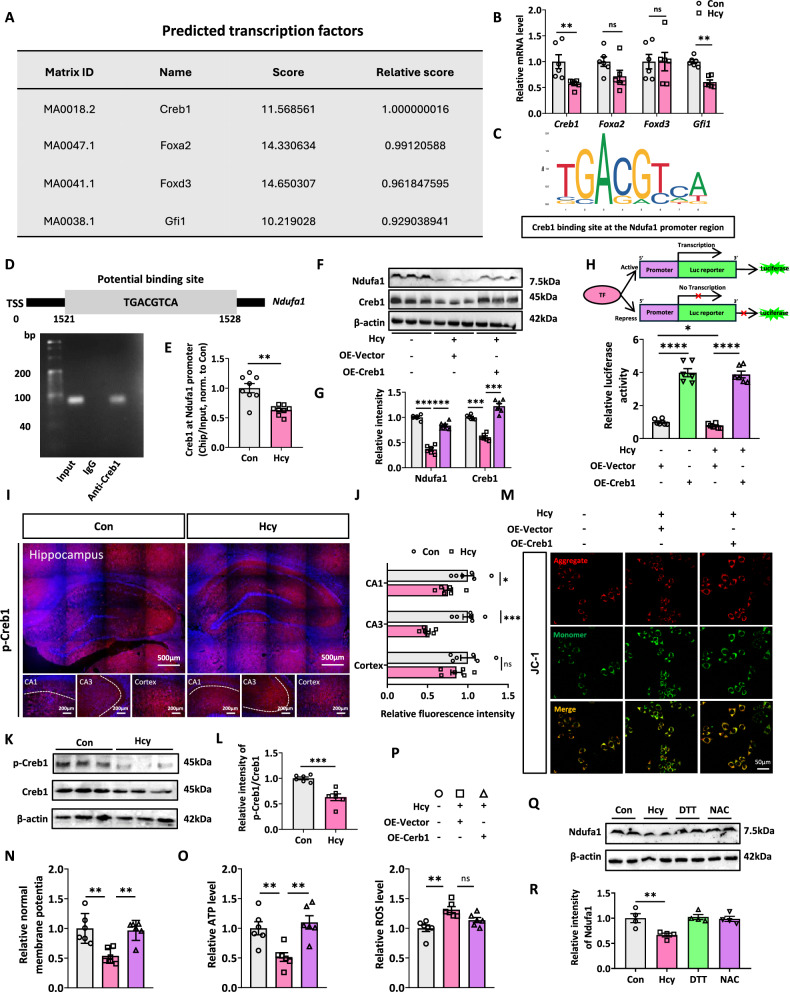
Hcy decreases the expression level of Ndufa1 by reducing the expression of the transcription factor Creb1*.* **A** Transcription factor prediction of Ndufa1 was performed by NCBI (https://www.ncbi.nlm.nih.gov/home/download/) and Jaspar (https://jaspar.elixir.no/). The list showed the top 4 candidate genes overall. **B** RT-qPCR analysis of transcript levels of *Creb1*, *Foxa2*, *Foxd3*, and *Gfi1* in the hippocampus of rats treated with or without Hcy. The data were normalized by Gapdh (*n* = 5 for each group). **C** Potential locations of Creb1 binding to Ndufa1 predicted by JASPAR. **D**, **E** The ChIP-qPCR data of the Ndufa1 potential binding site in the promoter region with Creb1 in the hippocampus of rats (*n* = 4 for each group, each sample was repeated once). **F** Hcy reduced the protein levels of Ndufa1 and Creb1, and the overexpression of Creb1 reversed the reduction in HEK293T cells (*n* = 3 for each group). **G** Working principle of the dual-luciferase reporter gene. **H** Overexpression of Creb1 and a dual-luciferase reporter in which Ndufa1 promoter truncations were cloned and fused enhances transcription from the 5′ flanking region of the Ndufa1 gene. **I**, **J** p-Creb1 staining in CA1 and CA3 regions of the hippocampus and cortex in Con and Hcy groups. **L**, **M** The images of p-Creb1 and Creb1 by western blotting and the relative Creb1 activity were represented by the ratio of p-Creb1/Creb1. **K**, **N–P** JC-1 staining, ATP and ROS levels detection in N2a cells with/without Hcy and OE-Creb1 treatment. **Q**, **R** N2a cells were individually treated with Hcy (100 μM), DTT (100 μM), or NAC (100 μM) for 24 h. Changes in Ndufa1 protein levels were determined by Western blotting (*n* = 4). Data were presented as mean ± SEM. Unpaired *t*-test for (**E** and **L**). One-way ANOVA followed by Bonferroni’s post hoc was used for others (*P < 0.05, **P < 0.01, ***P < 0.001, ****P < 0.0001; ns no significance; *n* = 6 for each group).

To elucidate the molecular mechanism underlying the decrease in Ndufa1 induced by Hcy, we further investigated the transcriptional regulation of Ndufa1 expression by Hcy. We predicted the transcription factors regulating Ndufa1 through JASPAR (https://jaspar.genereg.net) and found that Creb1, Foxa2, Foxd3, and Gfi1 may be involved in the regulation of Ndufa1 expression (Fig. 8A). RT-qPCR revealed that the mRNA levels of Creb1 and Gfi1 were lower in the hippocampus of Hcy-treated rats than in those of Con rats (Fig. 8B). Since Gfi has been reported to be a transcriptional repressor, we chose Creb1 as a potential transcription factor regulating Ndufa1 [22, 44]. The binding of Creb1 to the Ndufa1 transcription promoter region was predicted by Jaspar and confirmed by a ChIP assay in the hippocampus of rats (Fig. 8C, D). We found that Creb1 was able to act on the potential binding site in the Ndufa1 promoter region predicted by JASPAR. Hcy treatment significantly reduced the affinity of Creb1 for the Ndufa1 gene promoter region (Fig. 8E). Furthermore, overexpressing Creb1 rescured the Hcy-induced decrease in Ndufa1 expression level (Fig. 8F, G). We also observed that Hcy exposure significantly decreased the luciferase activity of Ndufa1, and overexpression of Creb1 reversed the decreased luciferase activity of Ndufa1 induced by Hcy (Fig. 8H). These results suggested that Hcy reduced Ndufa1 gene transcription via mechanisms involving Creb1 inhibition.

To further investigate the expression of Creb1 in the hippocampus and cortex, p-Creb1 (Ser133) staining was used. The result of staining showed that the fluorescence intensity in CA1 and CA3 regions of the hippocampus of Hcy group was weaker than that of control group (Fig. 8I, J). In addition, the protein level of p-Creb1/Creb1 was decreased in HHcy rats by western blotting (Fig. 8K, L). This suggested that Hcy inhibited the activity of Creb1.

To further explore the role of the overexpression of Creb1 in Hcy-induced mitochondrial damage, we overexpressed Creb1 or Vector in N2a cells. JC-1 staining showed that overexpression of Creb1 significantly improved Hcy-induced MMP loss (Fig. 8M, N). Meanwhile, the level of ATP inhibited by Hcy was also restored after Creb1 treatment (Fig. 8O). Nevertheless, ROS levels did not significantly change between Hcy+OE-Vector and Hcy+OE-Creb1 groups (Fig. 8P). The above data indicated that overexpression of Creb1 could alleviate Hcy-induced mitochondrial function damage. Additionally, to verify whether the inhibition of Ndufa1 induced by Hcy is caused by metabolic changes or its thiol groups. We analyzed the effects of Dithiothreitol (DTT) or N-acetylcysteine (NAC) on Ndufa1 levels and found that neither of them could affect Ndufa1 expression in N2a cell (Fig. 8Q, R). These data indicated the inhibition of Ndufa1 induced by Hcy may be related to the metabolic changes, but not its thiol groups.

## Discussion

Mitochondrial defects are early pathological changes in neurodegenerative disease (ND). Homocysteine (Hcy) is an independent risk factor for ND. However, whether and how Hcy induces mitochondrial defects in the process of neurodegeneration is unclear. In this study, we identified that Hcy interfered with mitochondrial OXPHOS by inhibiting complex I, with upregulation of ROS in the hippocampus. Further molecular mechanism exploration indicated that Hcy suppressed the Ndufa1 expression, which is essential for complex I assembly and activation, through intervening in its transcription factor Creb1. In addition, we found Hcy-induced neurodegeneration-like pathology changes of mitochondria via inhibiting the NAD^+^/Sirt1 pathway, including defects in mitochondrial morphology, mitochondrial biogenesis, and mitophagy, ultimately leading to impairments of synapse and cognition. Finally, we demonstrated that Ndufa1 upregulation or NAD^+^ supplementation could reverse Hcy-induced complex I activity decline, mitochondrial homeostasis defects, and neurodegeneration. Thus, Ndufa1 is a key molecular switch of Hcy-induced mitochondrial damage, and targeting Ndufa1 or NAD^+^ replenishment appropriately can ameliorate Hcy-induced neurodegeneration and cognitive impairment.

Hcy is an intermediate product of the sulfur-containing amino acid and methionine cycle, which is maintained at normal levels in the body by remethylation to methionine in reactions that require dietary folic acid, vitamin B6, and B12 [45]. Hcy significantly leads to nerve damage in healthy individuals and exacerbates neurodegeneration in AD patients [46, 47]. Elevated plasma Hcy levels are associated with hippocampal atrophy, increased accumulation of Aβ, and enhanced tau phosphorylation, which are key pathological features of AD [48–50]. It is worth noting that for every 5 μM increase in Hcy, the risk of AD increases by 40%, and AD patients typically exhibit Hcy levels ≥15 μM [51, 52]. Hcy can also impair cognitive abilities, underscoring its crucial role in both initiating and exacerbating neurodegenerative processes [53]. Since Hcy plays a critical role in neurodegeneration, the exact mechanisms are unclear. To better understand the functional changes in the hippocampus of Hcy-treated rats, we conducted whole-hippocampus RNA sequencing on Con and Hcy groups. The transcriptomic data indicated that transcriptomic changes in mitochondrial genes were prominent features in the hippocampus of Hcy-treated rats, and defects in mitochondrial functions may specifically be involved. Mitochondrial dysfunctions are early pathology and pathophysiology changes in ND, including AD [2]. In addition to reducing cellular ATP levels, mitochondrial deficiencies facilitate the production of MitoSOX and intracellular ROS, which have the potential to cause harm to nucleic acids, proteins, and membrane lipids [54]. Importantly, damaged mitochondria can reduce membrane potential and increase mitochondrial membrane permeability. Caspase-3-dependent apoptosis is induced by the release of pro-apoptotic factors, including cytochrome C, apoptosis-inducing factor, and Smac, into the cytosol, a consequence of increased mitochondrial membrane permeability. Thus, the maintenance of healthy mitochondria is necessary for the functions and health of neurons. In the present study, we found that Hcy may induce defects in mitochondrial ultrastructure and ATP production, OXPHOS, complex I activity, and MMP. Recent studies found that mitochondrial hypermetabolism precedes impaired autophagy and synaptic disorganization in AD mice [55], and glucose hypermetabolism played a critical role in cellular aging [56], which is consistent with our observation that an increase in glucose uptake in Hcy-treated rats was measured by PET. These results indicated that mitochondria, as the critical target organelle of Hcy, contributed to the process of neurodegeneration.

Mitochondria undergo steady modification for mitochondrial quality control through two opposing processes: mitochondrial biogenesis, which generates fresh, functional ones; and mitophagy, which eliminates impaired and superfluous mitochondria through the autolysosomal pathway [57, 58]. Mitochondrial biogenesis is regulated by different signaling pathways that promote the formation of mitochondria. It is a process that is regulated at the transcription level, a synchronized bi-genomic program comprised of nuclear-encoded mitochondrial genes as well as the thirteen mitochondrial-encoded genes [18]. PGC1 family members (PGC1α and PGC1β) can activate genes encoding proteins, such as NRF1 and TFAM, for the replication and transcription of mtDNA and mitochondrial proteins [59, 60]. Some studies have demonstrated mitochondrial biogenesis-associated genes such as PGC1α, TFAM, and NRF2 decreased in the postmortem brain of AD patients compared with age-matched control individuals [61]. While upregulation of PGC1α by lentiviral vector-hPGC1α injected into the brain could effectively inhibit AD progression [62]. On the other hand, mitophagy is necessary for normal cellular functions, and mitophagy dysfunction has been linked to aging, neurodegeneration, and aging-related disease [63]. Evidence demonstrates that lifestyle interventions and pharmacological agents aimed at stimulating mitophagy might protect neurons against dysfunction and degeneration [9]. In damaged mitochondria, the inner mitochondrial membrane depolarizes, which leads the protein PINK1 to recruit PARKIN to the outer mitochondrial membrane. PARKIN is activated by phosphorylation (ser65) by PINK1 and then ubiquitylates several proteins, including P62, which recruit the mitochondria to the LC3-positive autophagy pathway [64]. In the current study, we observed that Hcy could induce mitochondrial biogenesis and mitophagy defects in the hippocampus of rats and N2a cells. Further molecular mechanisms exploration indicated that Hcy-induced defects in mitochondrial homeostasis by inhibiting the PINK1/PARKIN/PGC1α pathway.

Sirtuins are a group of nicotinamide adenine dinucleotide (NAD^+^)-dependent deacetylases that can regulate several cellular processes such as transcription, mitochondrial function, energy metabolism, and aging [65]. Several studies indicate that the reduction of Sirt1 and mitochondrial Sirt3 may contribute to ND [66]. Evidence indicated that Sirt1 regulated multiple aspects of mitochondria, including mitochondrial biogenesis and mitophagy. Sirt1 played a key role in mitochondrial biogenesis by upregulating PGC1α expression [67]. Elevating Sirt1 or NAD^+^ levels could promote the accumulation of PGC1α in the nucleus, which results in the transcription of genes that are necessary for mitochondrial function and biogenesis. In addition, Sirt1 induces mitophagy through the deacetylation and activation of the mitophagy proteins, including PINK1, PARKIN, and LC3 [37, 39]. In the present study, we found that Sirt1 was involved in the mechanism underlying the Hcy-induced mitochondrial dysfunction via inhibiting NAD^+^ metabolism.

The redox cofactor NAD^+^ plays a key role in mitochondrial metabolism and is a substrate for signaling enzymes, including Sirt1. NAD^+^ regeneration depends on complex I (NADH ubiquinone oxidoreductase) activity [23, 24]. We found that Hcy could significantly inhibit complex I activity in vivo and in vitro. These data indicated that complex I may be an important target for Hcy-induced mitochondrial impairment and NAD^+^ reduction. The RNA sequencing data in the current study showed that the mRNA level of Ndufa1, which is essential for complex I assembly and activation, was decreased significantly in the hippocampus of HHcy rats. Ndufa1 is the accessory and non-catalytic subunit of NADH dehydrogenase, an enzyme that is part of the mitochondrial membrane respiratory chain. Its primary function is to facilitate the transfer of electrons from NADH to the respiratory chain [27]. Recently, the least absolute shrinkage and selection operator (LASSO) and support vector machine recursive feature elimination (SVM-RFE) analyses identified Ndufa1 as a candidate gene for predicting late-onset AD (LOAD) and mild cognitive impairment (MCI) [31]. The mRNA levels of Ndufa1 were reduced significantly in LOAD and MCI groups compared with controls [31]. However, the mechanism of Ndufa1 reduction and the role of Ndufa1 in cognitive impairment are unclear. In the current study, we found that Hcy could inhibit the Ndufa1 expression by downregulating its transcription factor Creb1, leading to mitochondrial dysfunction through repression of the NAD^+^/Sirt1 pathway in rat brains. Upregulation of Ndufa1 could effectively rescue Hcy-induced mitochondrial impairment.

Mitochondria play significant roles in neuroplasticity. It is reported that mitochondria could regulate the growth and differentiation of axons through buffering cytosolic Ca^2+^ [68]. Furthermore, the fine mitochondria are essential for the formation and maintenance of dendritic spines and synapses in the hippocampus [69]. On the other hand, studies of animal models and affected patients indicated that mitochondrial dysfunction contributes to synaptic impairment and neuronal degeneration in the brain, including AD [9]. In the current study, in addition to mitochondrial dysfunction, we also found that Hcy treatment reduced the number of spines in the quaternary dendritic branches, and NAD^+^ supplementation improved the reduction of Hcy-induced dendritic spine density. Furthermore, Hcy treatment significantly inhibited the expression of PSD95, Synapsin1, and several postsynaptic-related proteins, including GluN2A, GluN2B, and GluN1 in hippocampal extracts. After the upregulation of Ndufa1 or NAD^+^ supplementation-induced recovery of mitochondria, the decrease in the levels of synaptic-related proteins was partially recovered. These data indicated that upregulation of Ndufa1 or NAD^+^ supplementation could not only alleviate Hcy-induced mitochondrial damage but also protect Hcy-induced synaptic plasticity damage, which may be related to the protection of mitochondria by NAD^+^.

**Fig. 9:**
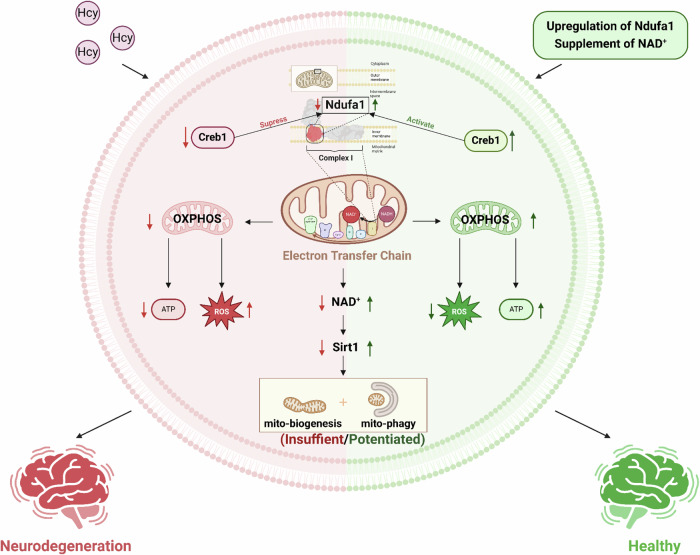
Hcy interferes with Ndufa1 leading to mitochondrial defects and neurodegeneration through repression of the NAD^+^/Sirt1 pathway in the brain. The graphical abstract was created with BioRender.com.

In sum, strong evidence has indicated that disturbed mitochondrial OXPHOS, biogenesis, and mitophagy are central pathological components underpinning neurodegenerative diseases. Our current findings indicated a possible link between Hcy and neurodegeneration: Hcy inhibits Ndufa1 expression, and loss of Ndufa1 disrupts the formation of complex I and thus interferes with mitochondrial biogenesis and mitophagy by repression of NAD^+^/Sirt1 pathway (Fig. 9), which eventually leads to impairments of synapse and cognition as seen in neurodegeneration. Ndufa1 upregulation or NAD^+^ supplement can normalize Hcy-induced mitochondrial homeostasis defects and cognitive impairment. Our study should provide important insight into the role of Hcy in the chronic course of neurodegeneration and mitochondrial homeostasis.

## Materials and methods

### Antibodies, siRNA, plasmids, and AAVs

The antibodies used in this study were shown in Table 1.

**Table 1:** Antibodies used in WB, IF, and Co-IP.

Antibody	Specificity	Type	Dilution for WB	Source	Catalog number
β-actin	β-actin	Poly-	1:1000	SAB	21335
AMPKa1	AMPK alpha 1	Mono-	1:1000	SAB	45527
p-AMPKa	Phospho-AMPK (Thr172)	Mono-	1:1000	SAB	48827
PGC1a	PGC-1 alpha	Mono-	1:1000 (1:100 for Co-IP)	Proteintech	1C1B2
m-PGC1a	Methyl-PGC1a (Lys224)	Poly-	1:1000	Affinity	AF8600
NRF1	NRF1	Poly-	1:1000	SAB	32900
TFAM	TFAM	Poly-	1:1000	SAB	29655
LC3B	LC3B	Poly-	1:1000 (1:100 for IF)	Abcam	45394
P62	SQSTM1	Poly-	1:1000	CST	23214
PINK1	PINK1	Poly-	1:1000	SAB	55131
PARKIN	Parkin	Poly-	1:1000	SAB	53224
TOMM40	TOMM40	Mono-	1:1000 (1:100 for IF)	Beyotime	AG4582
COX5A	COX5A	Mono-	1:1000	SAB	34224
SDHB	SDHB	Poly-	1:1000	SAB	41524
UQCRC2	UQCRC2	Poly-	1:1000	SAB	54535
ATP5A	ATP5A	Poly-	1:1000	SAB	40625
Sirt1	SIRT1	Poly-	1:1000	SAB	32029
p-Sirt1	Phosoho-Sirt1 (Ser47)	Poly-	1:1000	Bioss	Bs-3393R
Acetylated-Lysine	Acetylated-Lysine	Poly-	1:1000	CST	9441S
GluN2A	NMDAR2A	Poly-	1:1000	SAB	53091
GluN2B	NMDAR2B	Poly-	1:1000	SAB	54739
GluN1	NMDAR1	Poly-	1:1000	SAB	49488
SYN1	Synapsin-1	Poly-	1:1000	SAB	41470
PSD95	PSD95	Poly-	1:1000	SAB	45221
Iba1	IBA1	Mono-	1:100 for IF	Abcam	ab283319
Caspase3	Caspase3/p17/p19	Poly-	1:100 for IF	Proteintech	19677-1-AP
Ndufa1	NDUFA1	Poly-	1:1000	Proteintech	15561-1-AP
Creb1	CREB1	Poly-	1:1000	Proteintech	12208-1-AP
p-Creb1	Phosoho-Creb1 (Ser133)	Poly-	1:1000	SAB	13951

*WB* western blotting, *IF* immunofluorescence, *Co-IP* co-immunoprecipitation, *Mono-* monoclonal, *poly-* polyclonal.

The siRNA used in this study were shown as follows: siVector (negative control), Sense Strand: 5′-UUCUCCGAACGUGUCACGUTT-3′, Antisense Strand: 3′-ACGUGACACGUUCGGAGAATT-5′; si*Ndufa1*, Sense Strand: 5′-GAGUUGCUCGUGUUCAGUATT-3′, Antisense Strand: 3′-UACUGAACACGAGCAACUCTT-5′ were from Obio Technology (Shanghai, China).

The plasmids used in this study were shown as follows: pcDNA3.1–3 × Flag, pcDNA3.1-rNdufa1-3 × Flag-C, pcDNA3.1-rCREB1-3 × Flag-C, pDualuc-Basic, Ndufa1-pDualuc-Basic were from UNIBIO Biological Technology (Changsha, China).

The AAVs used in this study were shown as follows: pAAv-CMV-MCS-EF1a-EGFP-tWAP, pAAv-CMV-Ndufa1-3 × FLAG- EF1a-EGFP-tWAP were from Obio Technology (Shanghai, China).

### Animals

Two-month-old male Sprague-Dawley (SD) rats were purchased from SPF Biotechnology Company (Beijing, China). The animals were raised under conditions that met SPF standards, with temperatures ranging from 21 °C to 24 °C and relative humidities ranging from 40% to 60%. The animals were provided with a 12-h light and 12-h dark cycle, allowing unrestricted intake of food and water. The animal experiment plan was approved by the Experimental Animal Ethics Committee of Jiangnan University (reference number: JN. No20230615S0300815[320]).

To meet the needs of each experimental batch and ensure sufficient statistical power, we performed experiments using 12 rats per group (including 24 hippocampal tissues). The first batch involved phenotyping experiments (Hcy and Con groups). Within this batch, 3 rats were used for mRNA sequencing, 5 for TEM, 6 for IF, and the remainder for other molecular biology assays. The second batch consisted of AAVs experiments (Hcy+AAV-Vector, Hcy+AAV-Ndufa1, and Con groups). Within this batch, 8 rats underwent behavioral tests (all animals were collected and tissues sampled after the behavioral experiments), 5 were used for Golgi staining, 6 for IF, and the remaining animals were used for other molecular biology assays. The third batch comprised NAD^+^ treatment experiments (Hcy+NAD^+^, Hcy, and Con groups). Within this batch, 8 rats underwent behavioral tests (all animals were collected and tissues sampled after behavioral experiments), 5 were used for Golgi staining, 6 for IF, and the remaining rats were used for other molecular biology assays.

Rats were randomly assigned to different groups using a computer-generated random number sequence in Microsoft Excel. Each rat was assigned a unique identification number, and a random number was generated for each rat using the RAND function. Rats were then sorted based on their random numbers. Data from all rats initially included in the study were analyzed, except for those that died due to reasons independent of the experimental treatment (such as accidental death during behavioral test). The double-blind method was used for animal experiments in this study.

### Hcy modeling, drug treatment, and brain stereotaxic injection

The HHcy model was established by tail vein injection of 400 μg/kg/d DL-homocysteine (STBJ6336, Sigma, USA) saline solution for 3 weeks [70]. The Hcy+NAD^+^ group received the same treatment in addition to daily intraperitoneal injections of 500 mg/kg/d NMN (C15175108, Macklin, China) in saline solution for 3 weeks [71].

AAVs were purchased from Obio Biotech (Shanghai, China). The rats were anesthetized via the intraperitoneal injection of 20% urethane saline solution (800 mg/kg). AAVs (10^12^ IU/ml, 2 μl) for Ndufa1 overexpression and the control viruses were bilaterally microinfused into the hippocampus with a stereotactic injector (Stoelting CO. 620, USA). Holes were drilled above the CA3 field of the hippocampus (bregma: anterior/posterior −2.0 mm, medial/lateral −1.5 mm, and dorsal/ventral −2 mm). The injection speed was 0.50 μl/min. After injection, the microsyringe needle was left in the brain for 3 min [72]. Hcy treatment was performed from the second week after AAVs injection. Behavior tests and tissue extraction were carried out after the completion of Hcy tail vein injection.

### Novel object recognition (NOR)

The animals were placed in the laboratory for 24 h before the test began. One day before the beginning of the test, the rats were given 5 min to become accustomed to an empty arena (a 50 × 50 × 50 cm^3^ plastic container). During the first day of training, two objects (A and B) were placed at opposite corners of the arena and were positioned 3 cm away from the box’s inner wall. The rats were placed inside the box facing the inner wall and each rat was given 10 min to investigate. The next day, object B was swapped for a novel object C, which was of the same material and size but different in shape. Between each habituation period, 70% ethanol was used to clean the area and objects. A camera system was directly positioned above the box to record. Object exploration was quantified as the time it took the rat to smell, lick, or touch the object using its head or front paws [73].

### Morris water maze (MWM)

MWM was used to measure the spatial learning and memory of the animals. A circular pool (135 cm diameter, 60 cm height) was filled to a depth of 40 cm with water at 21–24 °C, and the pool was divided essentially into 4 equal quadrants. Black ink was added to make the water opaque. A transparent platform (10 cm × 5 cm) was placed in the fourth quadrant and submerged 1–2 cm below the water’s surface.

During each training session on days 1–6, the rats were randomly placed into different quadrants facing the inner wall of the pool and given 60 s to find and climb onto the platform. If the rats could not find the platform within 60 s, the experimenter guided them to the platform. The rat was allowed to stay on the platform for 30 s each round to ensure it remembered where it was. Each rat was trained for 4 rounds per day. The time elapsed before reaching the platform was recorded as the latency period. On day 7, the rats were tested and allowed to search for the position of the platform within 60 s. A camera was placed 1.5 m above the water surface, and the pool was surrounded by curtains and LED tubes to provide ample lighting. Escape latency and path length during each trial were recorded using a computerized digital tracking system (WMT-100S TECHMAN, China).

### Fear condition test (FCT)

The day before the test, animals were placed in the fear condition chamber (Taimeng FCT100, China), with the first 3 min as the adaptation period. During the training phase, a noise stimulation (2 kHz, 80 dB, 28 s) and a foot shock (1 mA, 2 s) were administered, a total of three shocks, with a time interval of 30 s between each two shocks. 2 h and 24 h later, rats were placed in the shock chamber for 3 min without receiving a shock. If the rats remembered the association of the chamber with the previous shock, they exhibited a fear response characterized by freezing behavior. Freezing time was automatically recorded by the software. The chamber was cleaned with 75% ethanol after each trial to minimize olfactory cues that could influence the behavior of subsequent rats.

### PET/CT imaging

The assessment of the biological response was evaluated by a small animal micro-PET scanner (Pingseng Healthcare Co., Ltd., Suzhou, China) using [^18^F] FDG. Six rats were separated into two groups (Hcy and Con groups, *n* = 3 in each group). [^18^F] FDG scans were performed after the tail vein injection of Hcy (400 μg/kg/d) or PBS. A 30-gauge needle was inserted into the tail vein, enabling the injection of the radioactive tracer ([^18^F] FDG dissolved in 200 μL saline). The rats were anesthetized with 1.5–2% isoflurane in 0.5 L/min air flow and then subjected to dynamic imaging for 30 min. Static imaging was performed at 90 min. The obtained images were reconstructed by using three-dimensional ordered-subset expectation maximization and the point spread function with the attenuation correction of CT and subsequently processed by using PMOD (version 4.3, PMOD Technologies). Regions of interest (ROIs) were drawn on brain images, and corresponding signal levels were measured. Delineation of the brain region and data analysis were conducted by PMOD (V3.5, PMOD Technologies, USA).

### Electron transmission microscopy (TEM)

Ultrastructural analysis of the hippocampus was performed using TEM (HITACHI HT7650, Japan). Following completion of the behavior tests, rats were euthanized, and their hearts were perfused with a fixative solution containing 3% paraformaldehyde and 2% glutaraldehyde in PBS. Serial ultrathin sections (60–70 nm) from the hippocampus were then prepared and processed for TEM by Shiyanjia Lab (https://www.shiyanjia.com). The standards for the evaluation of mitochondrial injury were as previously described [74]. 6 serial sections from 5 rats per group were analyzed.

### Golgi staining

Fresh rat brains were washed in PBS (BL302A, Biosharp, China). The following staining steps were referenced to the Rapid GolgiStain kit (FD Neuro Technologies PK40, USA) instructions. Brains frozen with liquid nitrogen were cut into 100 μm slices using a cryotome (Leica CM3050S, USA). Slices were dried in the dark for 48 h, dehydrated through 85%, 95%, and 100% ethanol, cleared in xylene and coverslipped. Images were taken under a light field (Axio Imager Z2 Carl Zeiss, DE). Different spine shapes were categorized as described earlier [75], including mushroom and thin. For each experimental group, 10 neurons derived from 5 rats were counted to analyze the dendritic spine number in the hippocampus.

### mRNA sequencing and bioinformatics analysis

Transcriptome analysis was performed on unilateral hippocampal tissues from 3 rats each in the Con and Hcy groups. As previously mentioned [76], samples were subjected to RNA extraction and purification, reverse transcription, library construction, and sequencing at Mayo-Biopharmaceutical Biotechnology (Shanghai, China) according to the instructions of Illumina, Inc. (San Diego, USA). An Illumina TruSeq^TM^ total RNA sample preparation kit (20040534) was used to prepare the transcriptome library. The subsequent outcome data analysis steps are available online (https://www.majorbio.com).

Differentially expressed genes (DEGs) were screened according to *P*-value < 0.05 [77–79]. Cluster analysis of differentially expressed proteins and proteins was analyzed using the majorbio platform (https://www.majorbio.com). The functions of the DEGs were analyzed with the Functional Annotation Tool of DAVID Bioinformatics (https://davidbioinformatics.nih.gov/summry.jsp). Heat maps were produced using the Wei Sheng Xin platform (https://www.bioinformatics.com.cn) and GraphPad Prism 8.0. Protein-protein interaction analysis was retrieved using STRING 11.5 software and then visualized with Cytoscape 3.8.2.

### Tissue extraction and frozen sectioning

The rats were anesthetized via intraperitoneal injection of 20% urethane (800 mg/kg). Following thoracotomy, a perfusion needle was inserted into the left apex of the heart. The blood was flushed with 0.5% sodium nitrite solution and subsequently, the tissues were fixed with 4% formaldehyde solution. The brains were then removed and further fixed for 48 h in 4% formaldehyde at 4 °C followed by cryoprotection in 15% (for 24 h), 20% (for 24 h), 30% sucrose (for 72 h) at 4 °C. After the brain was frozen in OTC, it was cut into 14 μm slices with a cryotome (Leica CM3050S, USA) and stored at −80 °C.

### Immunofluorescence

Following rewarming, brain sections were washed with PBS for 15 min (5 min × 3 times) and blocked in blocking buffer (10% goat serum and 0.5% Triton X-100 in PBS) for 1 h at room temperature. After the slices were incubated with primary antibodies overnight at 4 °C, they were washed with PBS for 30 min (10 min × 3 times). The next day, sections were incubated with secondary antibodies (Alexa Fluor secondary antibodies, Jackson ImmunoResearch, USA) at room temperature for 1 h. Then, the slices were washed with PBS and sealed with DAPI-containing sealing tablets. Images were taken using a Zeiss laser confocal fluorescence microscope (Carl Zeiss LSM880, DE).

### ELISA

The concentration of Pink1 or Sirt1 activity was determined by using a rat PTEN-induced kinase 1 (PINK1) or Sirtuin 1 (Sirt1) activity assay (Tongwei Biotechnology Company, China) according to the manufacturer’s instructions. Briefly, hippocampal tissues or cells were diluted and added to the pre-coated wells. Following incubation and washing steps, the substrate solution was added, and the reaction was stopped with the provided stop solution. Absorbance was measured at 450 nm using a microplate reader (BioTek Instruments, USA). A standard curve was generated using the provided standards, and the Pink1 or Sirt1 activity was calculated.

### RT-qPCR and mtDNA quantification

RNA was purified using Total RNA Isolation Reagent (BS258A, Biosharp, China) and converted to cDNA with a HipScript III RT SuperMix for qPCR (R32301, Vazyme, China) according to the manufacturer’s instructions. The PCR mixture contained 10 μl of SYBR Green PCR master mix, 2 μl of forward and reverse primers, and 1 μg of cDNA. The volume of each well was replenished to 20 μl with diethylpyrocarbonate (DEPC H_2_O). cDNA was quantified via RT-qPCR (Roche LightCycler 480 II, USA) with ChamQ Universal SYBR qPCR Master Mix (7E750E3, Vazyme, China). The results were calculated using the 2^−ΔΔCt^ method.

Total DNA was extracted using a Genomic DNA Rapid Extraction kit (D0065S, Beyotime, China) and mtDNA content was subsequently measured using RT-qPCR (R32301, Vazyme, China). Mitochondrial DNA (mtDNA) copy number was determined by quantitative real-time PCR as described [80], quantification of mtDNA was performed by use of the ratio of mitochondrial gene (Cytochrome b) to nuclear gene (β-actin). Other Genes expression levels were normalized to the signals of GADPH expression. The primers were listed in Table 2.

**Table 2:** Primers employed in RT-qPCR.

Gene	Forward primer (5′ → 3′)	Reverse primer (5′ → 3′)
*Gapdh*	AGGCTGTGGGCAAGGTCATC	TTCTCCAGGCGGCATGTCAG
*β-actin*	ACCCACACTGTGCCCATCTAC	TCGGTGAGGATCTTCATGAGGTA
*cytochrome b*	GCGTCCTTGCCCTATTACTATC	CTTACTGGTTGTCCTCCGATTC
*Sirt1*	TGCCATCATGAAGCCAGAGA	AACATCGCAGTCTCCAAGGA
*Ndufa1*	TGGTTCGAGATTCTCCCTGG	ACTGGTACTGAACACGAGCA
*Creb1*	AACAATGGTACGGATGGGGT	AGGACGCCATAACAACTCCA
*Foxa2*	CCTACGCCAACATGAACTCG	TGCCGGTAGAAAGGGAAGAG
*Foxd3*	TCATCAGCAACCGTTTTCCG	TCCGAAGCTCTGCATCATCA
*Gfi1*	TTCCAGCCTCAGATGACCAG	CTCCATTTTCGACTCGCCTG
*Chip-Ndufa1*	CACAACTTTGGTTGCCTCAAGTAAATC	AGCCCTATTGCATAAGTTATGTGGATG
*Il-1β*	ATGGCAACTGTTCCTGAACTCAACTG	CAGGACAGGTATAGGATTGGATGGG
*Il-6*	TCCATCCAGTTGCCTTCTCC	GGTCTTGGTCCTTAGCCACTC
*Tnfa*	CATCTTCTCAAAATTCGAGTGACAA	TGGGAGTAGACAAGGTACAACCC
*Inos*	GAGGAGGGAGACACTTTGAGA	GGCTTAGGTTGTCCTTTGTCAT

### Western blotting

Protein concentrations were detected using a BCA protein assay kit (P0012, Beyotime, China). Fresh tissues/cells were incubated with RIPA lysis buffer (1 mM PMSF) on ice for 30 min, lysed by sonication, and then centrifuged at 12,000 rpm for 10 min at 4 °C, after which the precipitate was discarded. Electrophoresis gels were prepared with a One-Step PAGE Gel Fast Preparation Kit (EC301-5, Vazyme, China). The protein content in all samples was 20 μg. After electrophoresis, the proteins were transferred from the gel to a nitrocellulose (NC) membrane. The membrane was then blocked with 5% skim milk powder (P0216, Beyotime, China) or bovine serum albumin (BSA) (ST023, Beyotime, China) for 1 h. Then, the appropriately diluted primary antibody was incubated with the NC membrane overnight at 4 °C to allow specific binding to the target protein. The next day, the membranes were washed with TBST (Tris-buffered saline with Tween 80) solution. After washing, the samples were incubated with secondary antibodies of the same species as the primary antibody for 1 h at room temperature. Finally, blotting was performed via an imager (Tanon-5200MULTI, USA) and an enhanced ECL chemiluminescence detection kit (E41104, Vazyme, China). Images were analyzed with ImageJ software to determine gray values and quantify protein expression levels. Relative quantification was calculated based on the expression of β-actin as the internal control.

### Coimmunoprecipitation (Co-IP) assay

Co-IP was performed by using an Immunoprecipitation (IP) Kit (Protein A/G Agarose) (P2197S, Beyotime, China). In brief, hippocampal tissues were lysed in ice-cold RIPA lysis buffer (1 mM PMSF) and homogenized (60 Hz, 90 s) thoroughly. The lysates were then centrifuged at 14,000 × *g* for 15 min at 4 °C, and the supernatant was collected.

Equal amounts of anti-PGC1α were incubated with Protein A + G Agarose for 1 h at room temperature with gentle rotation. The beads were then removed by centrifugation at 1000 × *g* for 5 min. The pre-cleared lysate was incubated overnight at 4 °C with anti-PGC1α antibody (in Table 1) or control IgG as a negative control.

Following incubation, Protein A + G Agarose was washed three times with ice-cold PBS. The immunoprecipitated proteins were eluted by boiling the Protein A + G Agarose in SDS-PAGE (2×) loading buffer for 10 min. The samples were then subjected to SDS-PAGE and Western blotting analysis.

### Chromatin immunoprecipitation (ChIP)

The experimental procedures followed the instructions of the ChIP assay kit (P2078, Beyotime, China). Briefly, hippocampal tissues (25–30 mg) were cross-linked with formaldehyde for 15 min at room temperature. The cross-linking reaction was stopped by adding 1.1 ml glycine (10×) and rotated at 4 °C for 10 min and then centrifuged for 5 min. The tissues were washed twice with cooled PBS (1 mM PMSF). After adding 1–2 ml RIPA (1 mM PMSF), tissues were sonicated on ice using an ultrasound device (IID, Scientz, China) with sonicate for 20 s and pause for 30 s (15 cycles) to achieve an average fragment size of approximately 0.8–1.5 kb. Any remaining cellular debris was removed by centrifugation. Then, the supernatant was diluted with ChIP buffer, and agarose protein A + G Agarose/Salmon sperm DNA was used to minimize nonspecific binding. After centrifugation, the samples were incubated overnight at 4 °C with the addition of anti-PGC1α or anti-IgG according to the antibody manufacturer’s instructions. After that, the samples combined with the primary antibody were centrifuged. Three different buffers were used (low-salt immune complex wash buffer, high-salt immune complex wash buffer, and lithium chloride immune complex wash buffer) to wash the sediment. Lastly, the samples were washed once with TE (50 mM Tris HCl and 10 mM EDTA). Subsequent purification was performed via a DNA purification kit (D0033, Beyotime, China). DNA fragments were quantified by RT-qPCR (Roche 480, USA).

### Dual luciferase assay

The Dual-Lumi Luciferase Assay was performed with a kit (RG088S, Beyotime, China) according to the manufacturer’s protocol. Youbao Biotechnology Company (Changsha, China) synthesized a dual-luciferase reporter vector specifically designed to analyze the role of the transcription factor Creb1 binding site in the Ndufa1 promoter. This vector incorporates a fragment of the Ndufa1 promoter region containing the binding site: TGACGTA (5′-3′), driving expression of the firefly luciferase reporter gene. HEK293T cells were co-transfected with a dual-luciferase reporter plasmid and either an overexpression Creb1 plasmid or an overexpression negative control plasmid. 48 h later, cells were treated with either Hcy (100 μM) or PBS for 24 h. The fluorescence intensity was determined with a microplate reader (Synergy H4, USA). Each experiment was repeated at least 3 times. The luciferase activity of each sample was normalized against Renilla.

### Cell culture and treatment

N2a cells (Procell Biological Technology Company, CL0168, China) and HEK293T cells (ATCC, CRL-3216, USA) were purchased and confirmed to be free of mycoplasma contamination. The cells were incubated in high-sugar medium (DMEM/high-glucose) (SH30243.01, Cytiva, USA) supplemented with 10% fetal bovine serum (FBS) (BS1614-109, Bioexplorer, USA) and 1% penicillin-streptomycin solution (BL505A, Biosharp, China) in a sterile incubator at 37 °C and 5% CO_2_.

Transfection of plasmids in cells was carried out with Lipofectamine™ 2000 Transfection Reagent (11668019, Thermo Scientific, USA) by following the manufacturer’s instructions. Cells were stimulated with 100 μM Hcy for 24 h and/or transfected with plasmids for 6–24 h. 100 μM DL-dithiothreitol (DTT) (HY-15917, MedChemExpress, China) or 100 μM Acetylcysteine (NAC) (HY-B0215, MedChemExpress, China), dissolved in DMSO (0.1%), was used to treated N2a cells for 24 h. All reagents were sterile, and all operations were completed in a biosafety cabinet.

### Mito-tracker and Lyso-tracker staining

N2a cells were prepared in confocal Petri dishes. When confluence reached 70–80%, cells were stained with MitoTracker (100 nM) (40743ES50, YEASEN, China) and LysoTracker (100 nM) fluorescent probes (40738ES50, YEASEN, China) for 45 min in the dark at 37 °C. Images were taken using a laser confocal fluorescence microscope (LSM880, Carl Zeiss, Germany). The co-localization of lysosomes and mitochondria was analyzed using ImageJ software.

### Mitochondrial membrane potential (MMP) detection

JC-1 kit (C2006, Beyotime, China) was used to assess the MMP level of cells according to the manufacturer’s protocols. Briefly, N2a cells were seeded at a density of 1 × 10^5^ cells/dish in confocal microplates. Cells were transfected with the indicated plasmids for 72 h or treated with Hcy (100 μM) for 24 h after plasmid transfection for 48 h. JC-1 staining solution (diluted to 1× by JC-1 staining buffer) was added to the cells, and incubated at 37 °C for 30 min. Two washes with staining buffer were followed by the laminar scanning of the cells with a Zeiss laser confocal fluorescence microscope (Carl Zeiss LSM880, DE).

### Measurement of ATP level

The ATP level was measured with an ATP detection kit (S00266, Beyotime, China). Briefly, tissue/cell samples were mixed with ATP detection fluid and incubated at room temperature for 3 min after protein extraction and measurement, after which the contents were read with a microplate reader. The ATP content was read by a microplate reader (Synergy H4, USA) with luminometer function.

### Measurement of intracellular and mitochondrial ROS

The generation of ROS was determined with a reactive oxygen species (ROS) assay kit (E004, Nanjing Jiancheng Bioengineering Institute, China). The assay employs DCFH-DA, which permeates the cell membrane and is hydrolyzed to DCFH monomers. These monomers are subsequently oxidized by ROS to form the fluorescent DCF, retained within the cells. Tissues or cells were loaded with the DCFH-DA fluorescent probe, incubated at 37 °C for 20 min in the dark, and then washed with serum-free medium for 5 min. Fluorescence intensity, indicative of ROS levels, was measured using flow cytometry (BD FACSAriall, USA) or the microplate reader (Synergy H4, USA).

MitoSOX red (441955 A, Tongwei Biotechnology Company, China) dye is a living-cell permeant and is capable of selectively targeting mitochondria where once it was oxidized by superoxide, it would produce red fluorescence (λex = 510 nm; λem = 590 nm). MitoSOX red solution (1:200 dilution) was added to N2a cells and incubated at 37 °C for 15 min. Then, cells were washed with PBS for 3 times. A laser confocal fluorescence microscope (LSM880, Carl Zeiss, Germany) was used for observation and photo analysis.

### Measurement of MDA level

MDA content was determined via an MDA detection kit (S0131S, Beyotime, China) according to the manufacturer’s instructions. The amount of MDA was measured by the reaction of one molecule of MDA with two molecules of thiobarbituric acid (TBA) to yield a pink-colored chromogen. The color reaction was measured at 532 nm with a reference wavelength of 450 nm by a microplate reader (Synergy H4, USA).

### Measurement of SOD activity

SOD activity was detected via a total SOD activity detection kit (S0101, Beyotime, China) by the reaction of NBT (nitro blue tetrazolium) with two molecules of superoxide anion to yield a blue-colored chromogen. SOD can inhibit the superoxide anion-free radical O^2−^. According to the manufacturer’s instructions, the absorbance was read at 560 nm by a microplate reader (Synergy H4, USA).

### Measurement of NAD^+^/NADH

NAD^+^/NADH levels were detected using a NAD^+^/NADH detection kit (WST-8) (S0175, Beyotime, China). The amount of formazan generated in the reaction system is proportional to the total amount of NAD^+^ and NADH in the sample. After heating in a 60 °C water bath for 30 min, the NAD^+^ in the sample decomposed, and only NADH was retained. NADH reduces WST-8 to formazan, and the amount of formazan generated by the reaction is determined by colorimetry, which ultimately determines the amount of NADH in the sample. Based on the total amount of NAD^+^ and NADH obtained from the first two steps of detection, the amounts of NADH and NAD^+^ and the ratio of NAD^+^/NADH in the sample could be calculated.

### Mitochondrial ETC complex I activity assay

A mitochondrial ETC complex I/NADH-CoQ reductase activity detection kit (BC0510, Solarbio, China) was used to determine complex I activity according to the manufacturer’s protocols. Briefly, hippocampal tissues or cells were homogenized to extract complex I, and the consumption of 1 nM of NADH mg protein/min was defined as an enzyme activity unit. The OD value was measured by using a microplate reader (Synergy H4, USA) for quantification.

### OCR determination

The oxygen consumption rate (OCR) was determined using a Seahorse XF96 (Agilent Technologies, USA) according to the manufacturer’s instructions and a Seahorse XF Cell Mitochondrial Stress Test Kit (103010-100, Agilent Technologies, USA). HEK293T cells were cultured in Seahorse cell culture plates in a CO_2_ incubator at 37 °C. After treatment (100 μM Hcy, 24 h), the medium was changed to unbuffered XF assay medium (103793, Agilent Technologies, USA) supplemented with 5 mM glucose, 1 mM pyruvate, and 2 mM glutamine and incubated in a CO_2_-free incubator at 37 °C for 1 h. Then, the cells were treated consecutively with oligomycin (an ATP synthase inhibitor, 1.5 μM), FCCP (carbonyl cyanide-4-(trifluoromethoxy) phenylhydrazone, an uncoupling agent, 2 μM), and antimycin A and rotenone (complex III and I inhibitors, respectively, 0.5 μM) every 20 min. Oligomycin inhibits mitochondrial ATP synthase activity and facilitates the measurement of ATP-linked respiration. FCCP uncouples the mitochondrial proton gradient and disrupts the mitochondrial membrane potential allowing measurement of maximal respiration. Rotenone inhibits mitochondrial complex I and antimycin inhibits mitochondrial complex III. The use of rotenone and antimycin together shut down mitochondrial respiration and allows measurement of non-mitochondrial respiration.

### Statistical analysis

The data were expressed as the mean ± SEM. All statistics presented in the article were analyzed and drawn using GraphPad Prism software 9 (La Jolla, CA, USA). Statistical significance was determined using unpaired *t*-tests, one-way ANOVAs, and two-way ANOVAs, followed by Bonferroni’s post hoc test when the variances were equal. *P* < 0.05 was considered statistically significant.

## Supplementary information

Supplementary data

Original data

## Data Availability

All data used to support the findings of this study are included within the article, and raw data is available from the corresponding author.
