# Supplementary material for: Homocysteine interferes with Ndufa1 leading to mitochondrial dysfunction through repression of the NAD+/Sirt1 pathway in the brain: a possible link between hyperhomocysteinemia and neurodegeneration
Source: Cell Death Dis. 2025 Jul 7;16(1):499. doi: 10.1038/s41419-025-07834-3 (PMC12234694; doi:10.1038/s41419-025-07834-3)
Supplement: Supplementary file 1 — Supplementary data [file 41419_2025_7834_MOESM1_ESM.docx]

**Supplementary data**

**
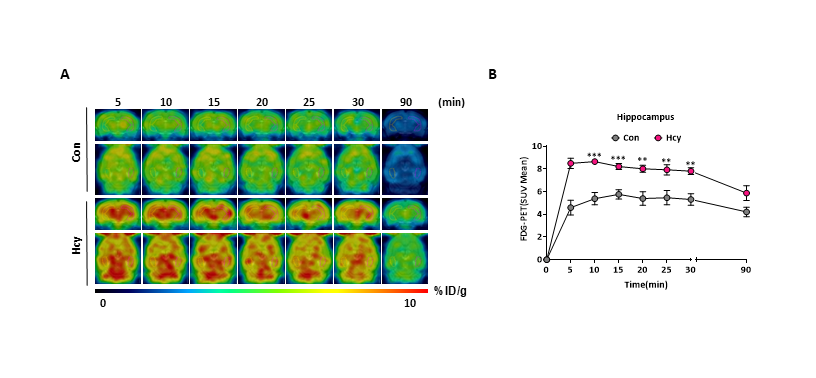
**

**Fig. S1 Hcy induced an increase in glucose uptake in the hippocampus of rats.** (**A**) Mean glucose uptake in the hippocampus of rats between Con and Hcy groups at different time points. Warm colors represented tissues with high glucose intake, while cool colors represented tissues with low glucose intake. The areas outlined in red and yellow were the bilateral hippocampal areas of the rats. PET/CT imaging and biodistribution analysis of ^18^F-FDG in the brains of rats treated with or without Hcy. (**B**) showed the values of glucose intake at different time points in the hippocampus of rats. Data were presented as mean ± SEM. Two-way repeated measures ANOVA followed by Bonferroni’s post hoc test was used to analyze the data (***P* < 0.01, ****P* < 0.001; n = 3 for each group).

**
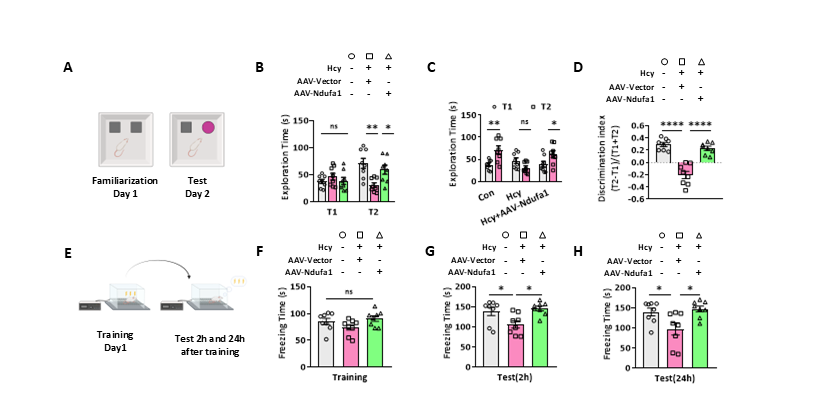
**

**Fig. S2 Upregulating Ndufa1 rescues Hcy-induced cognitive impairment in rats.** (**A**) NOR showed that the Hcy group had poor short-term memory ability, and upregulation of Ndufa1 rescued Hcy-induced cognitive impairment in rats. (**B**) Time spent familiarizing old and novel objects among 3 groups (T1: Time to identify old objects; T2: Time to identify novel objects). (**C**) Time to identify old and novel objects within each group. (**D**) The discrimination index was calculated by (T2-T1)/(T1+T2). (**E**) Fear conditional test showed that HHcy rats had short-term cognitive impairment. (**F**) Freezing time during conditional stimulus training. (**G**) Freezing time in test 2 h later. (**H**) Freezing time in test after 24 h. Data were presented as mean ± SEM. Two-way repeated measures ANOVA followed by Bonferroni’s post hoc test for C. One-way ANOVA followed by Bonferroni’s post hoc test for others (**P* < 0.05, ***P* < 0.01, *****P* < 0.0001; n = 8 for each group).

**
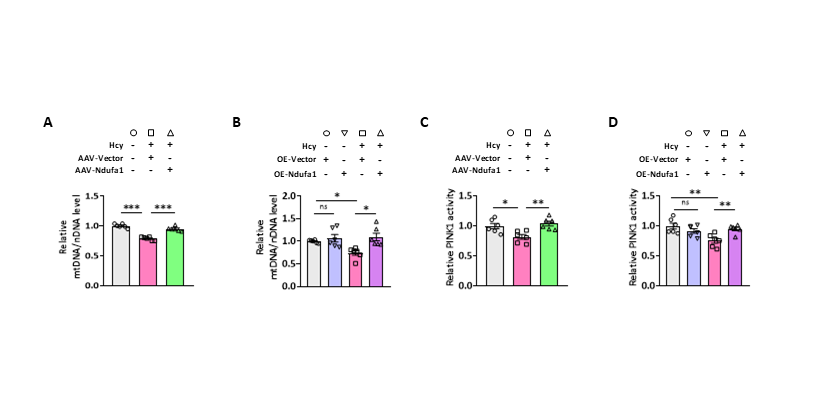
**

**Fig. S3 Upregulating Ndufa1 rescues Hcy-induced mito-biogenesis and mitophagy defects.** (**A, B**) Ndufa1 rescued the decreased mtDNA/nDNA levels caused by Hcy treatment in rats and N2a cells. (**C, D**) The activity of PINK1 was improved by Ndufa1 in HHcy rats and N2a cells. Data were presented as mean ± SEM. One-way ANOVA followed by Bonferroni’s post hoc test was used to analyze the data (**P* < 0.05, ***P* < 0.01, ****P* < 0.001; n = 6 for each group).


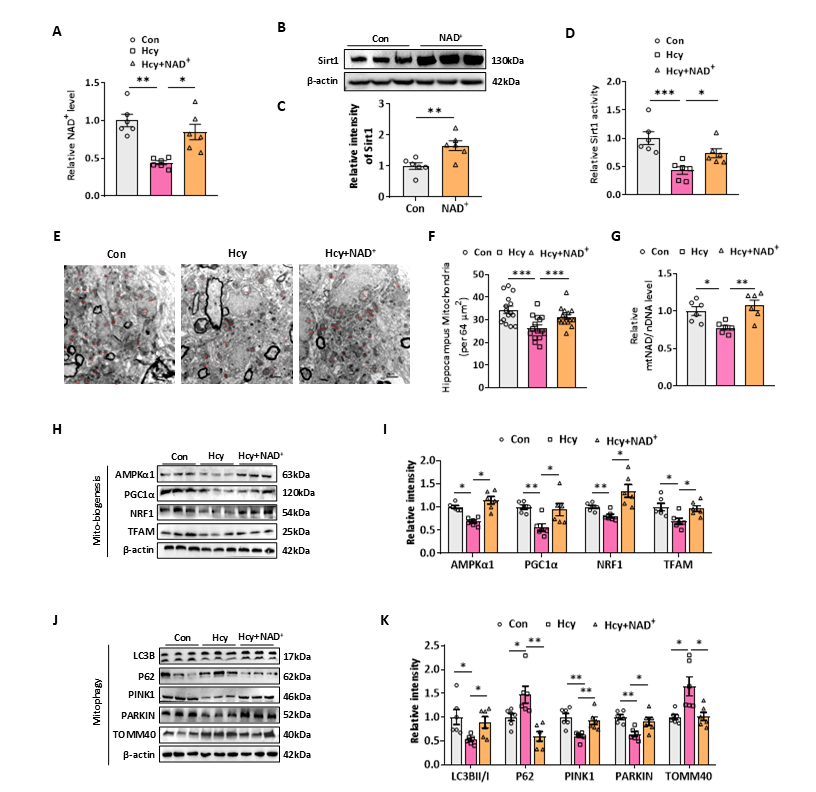


**Fig. S4 NAD^+^ supplementation ameliorates Hcy-induced mito-biogenesis and mitophagy dysfunction in rats.** (**A**) Relative NAD^+^ level was tested by the related assay. (**B-D**) Quantification of protein and activity levels of Sirt1. (**E, F**) Representative TEM images of mitochondria during Hcy and NAD^+^ treatment. The red “m” in the images represented mitochondria (n = 5 for each group, 6 serial sections per rat). (**G**) The mtDNA/nDNA was quantified by RT-qPCR. (**H, I**) SDS-PAGE and Western blotting analysis of AMPKα1, PGC1α, NRF1 and TFAM treated with or without Hcy and/or NAD^+^ in rats. (**J, K**) SDS-PAGE and Western blotting analysis of LC3B, P62, PINK1, PARKIN and TOMM40 treated with or without Hcy and NAD^+^ in rats. Data were presented as mean ± SEM. Unpaired t-test for C. One-way ANOVA followed by Bonferroni’s post hoc test for others (**P* < 0.05, ***P* < 0.01, ****P* < 0.00, ns: no significance; n = 6 for each group).
