# Supplementary material for: Homocysteine interferes with Ndufa1 leading to mitochondrial dysfunction through repression of the NAD+/Sirt1 pathway in the brain: a possible link between hyperhomocysteinemia and neurodegeneration
Source: Cell Death Dis. 2025 Jul 7;16(1):499. doi: 10.1038/s41419-025-07834-3 (PMC12234694; doi:10.1038/s41419-025-07834-3)

Original data for RT-qPCR

Homocysteine interferes with Ndufa1 leading to mitochondrial dysfunction through repression of the NAD^+ /Sirt1 pathway in the rat brain: A possible link between hyperhomocysteinemia and neurodegeneration

Source Data for Fig. 2C												
	c1	c2	c3	c4	c5	c6	h1	h2	h3	h4	h5	h6
nDNA	19.16	19.19	19.9	19.63	19.79	19.41	19.54	19.52	19.07	19.61	19.2	19.35
mtDNA	24.2	24.52	24.89	24.56	24.68	24.81	24.83	24.79	25.42	25.08	24.96	25.21
△ct	5.04	5.33	4.99	4.93	4.89	5.4	5.29	5.27	6.35	5.47	5.76	5.86
△△ct	-0.056667	0.233333	-0.106667	-0.166667	-0.206667	0.303333	0.193333	0.173333	1.253333	0.373333	0.663333	0.763333
2^-△△ct	1.040060	0.850667	1.076738	1.122462	1.154019	0.810378	0.874583	0.886791	0.419478	0.771997	0.631418	0.589134
Relative level	1.030728	0.843034	1.067076	1.112391	1.143664	0.803107	0.866735	0.878835	0.415714	0.765070	0.625752	0.583847

Source Data for Fig. 2U												
	c1	c2	c3	c4	c5	c6	h1	h2	h3	h4	h5	h6
Gapdh	18.24	18.27	19.06	19.22	19.35	18.29	18.86	18.24	18.03	18.11	19.24	19.35
Sirt1	28.36	28.45	28.90	28.96	29.30	28.52	29.75	28.66	29.08	28.76	29.85	30.46
△ct	10.12	10.18	9.84	9.74	9.95	10.23	10.89	10.42	11.05	10.65	10.61	11.11
△△ct	0.110000	0.170000	-0.170000	-0.270000	-0.060000	0.220000	0.880000	0.410000	1.040000	0.640000	0.600000	1.100000
2^-△△ct	0.926588	0.888843	1.125058	1.205808	1.042466	0.858565	0.543367	0.752623	0.486327	0.641713	0.659754	0.466516
Relative level	0.919336	0.881886	1.116253	1.196371	1.034307	0.851846	0.539115	0.746733	0.482521	0.636691	0.654591	0.462865

Source Data for Fig. 3D												
	c1	c2	c3	c4	c5	c6	h1	h2	h3	h4	h5	h6
Gapdh	16.88	19.75	18.66	17.98	15.78	15.78	15.3	19.33	18.01	18.54	15.1	19.85
Ndufa1	26.17	29.21	29	28.34	26.37	26.35	27.15	30.5	28.95	29.72	25.94	31.43
△ct	9.29	9.46	10.34	10.34	10.59	10.57	11.85	11.17	10.94	11.18	10.84	11.58
△△ct	-0.808333	-0.638333	0.241667	0.241667	0.491667	0.471667	1.751667	1.071667	0.841667	1.081667	0.741667	1.481667
2^-△△ct	1.751187	1.556530	0.845768	0.845768	0.711203	0.721131	0.296959	0.475769	0.557999	0.472483	0.598048	0.358075
Relative level	1.633675	1.452080	0.789013	0.789013	0.663478	0.672740	0.277031	0.443843	0.520555	0.440777	0.557917	0.334047

Source Data for Fig. 3H												
	c1	c2	c3	c4	c5	c6	h1	h2	h3	h4	h5	h6
Gapdh	16.87	19.75	18.66	17.99	15.78	15.78	15.29	19.33	18.01	18.54	15.1	19.85
Sirt1	26.77	30.12	28.59	27.69	25.49	26.39	26.88	29.99	28.25	29.59	26.49	31.1
△ct	9.9	10.37	9.93	9.7	9.71	10.61	11.59	10.66	10.24	11.05	11.39	11.25
△△ct	-0.136667	0.333333	-0.106667	-0.336667	-0.326667	0.573333	1.553333	0.623333	0.203333	1.013333	1.353333	1.213333
2^-△△ct	1.099362	0.793701	1.076738	1.262835	1.254112	0.672062	0.340722	0.649169	0.868541	0.495400	0.391387	0.431271
Relative level	1.071014	0.773234	1.048973	1.230272	1.221774	0.654732	0.331936	0.632430	0.846145	0.482626	0.381294	0.420150

Source Data for Fig. 3D												
	c1	c2	c3	c4	c5	c6	h1	h2	h3	h4	h5	h6
Gapdh	18.12	18.45	17.02	17.19	17.47	18.24	18.07	17.3	17.96	18.66	18.83	19.32
Sirt1	25.07	25.67	23.96	24.19	24.21	24.78	21.6	19.91	20.06	20.8	21.91	22.5
△ct	6.95	7.22	6.94	7	6.74	6.54	3.53	2.61	2.1	2.14	3.08	3.18
△△ct	0.051667	0.321667	0.041667	0.101667	-0.158333	-0.358333	-3.368333	-4.288333	-4.798333	-4.758333	-3.818333	-3.718333
2^-△△ct	0.964821	0.800145	0.971532	0.931956	1.115997	1.281944	10.326886	19.539658	27.825454	27.064566	14.106942	13.162242
Relative level	0.954261	0.791388	0.960899	0.921756	1.103783	1.267914	10.213861	19.325802	27.520913	26.768352	13.952545	13.018185

Source Data for Fig. 30

	c1	c2	c3	c4	c5	c6	H+Vec-1	H+Vec-2	H+Vec-3	H+Vec-4	H+Vec-5	H+Vec-6	H+Ndu-1	H+Ndu-2	H+Ndu-3	H+Ndu-4	H+Ndu-5	H+Ndu-6
Gapdh	19.65	20.33	20.83	20.9	20.96	21.15	19.35	19.46	19.68	19.99	20.31	20.48	19.61	20.32	20.45	20.52	20.6	20.86
Il-1β	25.48	25.62	25.77	26.03	26.1	26.43	23.55	23.76	23.83	23.86	24.11	24.18	24.89	24.98	25.31	25.46	25.5	25.52
Il6	26.32	26.38	26.52	26.66	26.71	27.8	23.89	23.88	23.92	24.12	24.56	24.63	25.43	25.51	25.67	25.72	25.97	26.04
Tnfa	28.39	28.5	28.53	28.67	28.77	28.81	25.62	26.68	25.71	26.76	26.83	26.53	25.78	26.83	26.9	27.35	27.69	27.42
Tnos	24.69	24.82	24.85	25.62	25.68	25.91	22.53	22.72	22.88	22.9	23.07	23.22	23.63	24.74	24.32	23.78	23.89	24.32
△ct	5.83	5.29	4.94	5.13	5.14	5.28	4.2	4.3	4.15	3.87	3.8	3.7	5.28	4.66	4.86	4.94	4.9	4.66
	6.67	6.05	5.69	5.76	5.75	6.65	4.54	4.42	4.24	4.13	4.25	4.15	5.82	5.19	5.22	5.2	5.37	5.18
	8.74	8.17	7.7	7.77	7.81	7.66	6.27	7.22	6.03	6.77	6.52	6.05	6.17	6.51	6.45	6.83	7.09	6.56
	5.04	4.49	4.02	4.72	4.72	4.76	3.18	3.26	3.2	2.91	2.76	2.74	4.02	4.42	3.87	3.26	3.29	3.46
△△ct	0.561667	0.021667	-0.328333	-0.138333	-0.128333	0.011667	-1.068333	-0.968333	-1.118333	-1.398333	-1.468333	-1.568333	0.011667	-0.608333	-0.408333	-0.328333	-0.368333	-0.608333
	0.575000	-0.045000	-0.405000	-0.335000	-0.345000	0.555000	-1.555000	-1.675000	-1.855000	-1.965000	-1.845000	-1.945000	-0.275000	-0.905000	-0.875000	-0.895000	-0.725000	-0.915000
	0.765000	0.195000	-0.275000	-0.205000	-0.165000	-0.315000	-1.705000	-0.755000	-1.945000	-1.205000	-1.455000	-1.925000	-1.805000	-1.465000	-1.525000	-1.145000	-0.885000	-1.415000
	0.415000	-0.135000	-0.605000	0.095000	0.095000	0.135000	-1.445000	-1.365000	-1.425000	-1.715000	-1.865000	-1.885000	-0.605000	-0.205000	-0.755000	-1.365000	-1.335000	-1.165000
2ˆ-△△ct	0.677519	0.985094	1.255562	1.100633	1.093030	0.991946	2.097009	1.956579	2.170960	2.635969	2.767021	2.965619	0.991946	1.524497	1.327152	1.255562	1.290861	1.524497
	0.671286	1.031683	1.324089	1.261377	1.270151	0.680657	2.938337	3.193194	3.617518	3.904127	3.592529	3.850378	1.209994	1.872544	1.834008	1.859610	1.652901	1.885569
	0.588453	0.873573	1.209994	1.152686	1.121166	1.244012	3.260289	1.687632	3.850378	2.305373	2.741566	3.797368	3.494292	2.760635	2.877867	2.211461	1.846765	2.666597
	0.750019	1.098093	1.520979	0.936272	0.936272	0.910670	2.722628	2.575763	2.685145	3.282966	3.642679	3.693529	1.520979	1.152686	1.687632	2.575763	2.522755	2.242332
Relative level	0.665999	0.968344	1.234213	1.081919	1.074445	0.975080	2.061353	1.923311	2.134047	2.591149	2.719972	2.915194	0.975080	1.498576	1.304586	1.234213	1.268912	1.498576
	0.645546	0.992123	1.273317	1.213010	1.221447	0.654557	2.825667	3.070751	3.478804	3.754423	3.454774	3.702735	1.163597	1.800742	1.763683	1.788303	1.589520	1.813267
	0.570402	0.846775	1.172876	1.117326	1.086773	1.205850	3.160275	1.635861	3.732261	2.234652	2.657464	3.680878	3.387099	2.675948	2.789584	2.143621	1.790112	2.584795
	0.731452	1.070909	1.483326	0.913094	0.913094	0.888125	2.655227	2.511998	2.618672	3.201694	3.552502	3.602093	1.483326	1.124151	1.645853	2.511998	2.460302	2.186821

Source Data for Fig. 8B

	c1	c2	c3	c4	c5	c6	h1	h2	h3	h4	h5	h6	n1	n2	n3	n4	n5	n6
Gapdh	18.85	18.9	18.92	21.71	21.78	21.81	18.61	18.82	19.7	19.72	19.78	20.88	17.82	17.84	18.86	19.01	19.14	20.5
Creb1	23.96	23.95	23.56	25.83	25.82	26.65	23.88	24.25	25.03	25.58	25.02	26.13	22.19	22.34	22.88	22.97	24.46	24.95
Foxa2	31.65	31.28	31.15	33.98	33.87	33.6	32.02	32.17	32.56	32.71	32.75	32.77	29.19	29.57	29.85	30.74	30.96	30.99
Foxd3	29.27	29.96	30	31.74	31.75	32.59	29.91	30.11	30.12	30.19	30.22	30.55	29.93	30.02	30.14	30.17	30.21	30.23
Gfi1	27.54	27.56	27.67	30.21	30.58	30.68	27.74	28.75	29.25	29.29	29.45	30.5	26.58	26.66	26.67	27.18	28.59	28.85
△ct	5.11	5.05	4.64	4.12	4.04	4.84	5.27	5.43	5.33	5.86	5.24	5.25	4.37	4.5	4.02	3.96	5.32	4.45
	12.8	12.38	12.23	12.27	12.09	11.79	13.41	13.35	12.86	12.99	12.97	11.89	11.37	11.73	10.99	11.73	11.82	10.49
	10.42	11.06	11.08	10.03	9.97	10.78	11.3	11.29	10.42	10.47	10.44	9.67	12.11	12.18	11.28	11.16	11.07	9.73
	8.69	8.66	8.75	8.5	8.8	8.87	9.13	9.93	9.55	9.57	9.67	9.62	8.76	8.82	7.81	8.17	9.45	8.35
△△ct	0.476667	0.416667	0.006667	-0.513333	-0.593333	0.206667	0.636667	0.796667	0.696667	1.226667	0.606667	0.616667	-0.263333	-0.133333	-0.613333	-0.673333	0.686667	-0.183333
	0.540000	0.120000	-0.030000	0.010000	-0.170000	-0.470000	1.150000	1.090000	0.600000	0.730000	0.710000	-0.370000	-0.890000	-0.530000	-1.270000	-0.530000	-0.440000	-1.770000
	-0.136667	0.503333	0.523333	-0.526667	-0.586667	0.223333	0.743333	0.733333	-0.136667	-0.086667	-0.116667	-0.886667	1.553333	1.623333	0.723333	0.603333	0.513333	-0.826667
	-0.021667	-0.051667	0.038333	-0.211667	0.088333	0.158333	0.418333	1.218333	0.838333	0.858333	0.958333	0.908333	0.048333	0.108333	-0.901667	-0.541667	0.738333	-0.361667
2ˆ-△△ct	0.718636	0.749154	0.995390	1.427344	1.508729	0.866537	0.643197	0.575678	0.616996	0.427304	0.656712	0.652176	1.200249	1.096825	1.529790	1.594753	0.621288	1.135504
	0.687771	0.920188	1.021012	0.993092	1.125058	1.385109	0.450625	0.469761	0.659754	0.602904	0.611320	1.292353	1.853176	1.443929	2.411616	1.443929	1.356604	3.410540
	1.099362	0.705475	0.695762	1.440597	1.501773	0.856584	0.597358	0.601513	1.099362	1.061914	1.084227	1.848899	0.340722	0.324585	0.605696	0.658231	0.700602	1.773583
	1.015132	1.036462	0.973779	1.158025	0.940609	0.896060	0.748289	0.429779	0.559289	0.551589	0.514651	0.532800	0.967053	0.927659	1.868223	1.455653	0.599431	1.284909
Relative level	0.688152	0.717375	0.953166	1.366798	1.444730	0.829779	0.615913	0.551258	0.590824	0.409178	0.628855	0.624511	1.149335	1.050299	1.464897	1.527105	0.594933	1.087337
	0.672940	0.900345	0.998996	0.971678	1.100799	1.355242	0.440908	0.459632	0.645527	0.589903	0.598138	1.264485	1.813216	1.412793	2.359613	1.412793	1.327351	3.336997
	1.047086	0.671929	0.662678	1.372094	1.430361	0.815852	0.568952	0.572910	1.047086	1.011418	1.032670	1.760981	0.324520	0.309150	0.576895	0.626931	0.667287	1.689246
	1.011748	1.033007	0.970533	1.154165	0.937474	0.893073	0.745794	0.428346	0.557425	0.549751	0.512936	0.531024	0.963830	0.924567	1.861996	1.450801	0.597433	1.280627

Source Data for Fig. S3A

	c1	c2	c3	c4	c5	c6	h+Vec-1	h+Vec-2	h+Vec-3	h+Vec-4	h+Vec-5	h+Vec-6	h+Ndu-1	h+Ndu-2	h+Ndu-3	h+Ndu-4	h+Ndu-5	h+Ndu-6
nDNA	16.87	19.75	18.66	17.99	15.78	15.78	15.29	19.33	18.01	18.54	15.1	19.85	19.16	16.06	15.91	15.92	16.52	17.62
mtDNA	26.82	29.7	28.68	28.01	25.78	25.86	25.59	29.75	28.32	28.88	25.4	30.27	29.14	26.14	25.98	25.99	26.66	27.74
△ct	9.95	9.95	10.02	10.02	10	10.08	10.3	10.42	10.32	10.34	10.3	10.42	9.98	10.08	10.07	10.08	10.14	10.12
△△ct	-0.053333	-0.053333	0.016667	0.016667	-0.003333	0.076667	0.296667	0.416667	0.316667	0.336667	0.296667	0.416667	-0.023333	0.076667	0.066667	0.076667	0.136667	0.116667
2ˆ-△△ct	1.037660	1.037660	0.988514	0.988514	1.002313	0.948246	0.814131	0.749154	0.802923	0.791869	0.814131	0.749154	1.016305	0.948246	0.954842	0.948246	0.909618	0.922316
Relative level	1.037157	1.037157	0.988035	0.988035	1.001828	0.947787	0.813737	0.748791	0.802534	0.791485	0.813737	0.748791	1.015813	0.947787	0.954379	0.947787	0.909178	0.921870

Source Data for Fig. S3B

	c+Vec-1	c+Vec-2	c+Vec-3	c+Vec-4	c+Vec-5	c+Vec-6	c+Ndu-1	c+Ndu-2	c+Ndu-3	c+Ndu-4	c+Ndu-5	c+Ndu-6	h+Vec-1	h+Vec-2	h+Vec-3	h+Vec-4	h+Vec-5	h+Vec-6
nDNA	20.03	20.09	20.23	20.28	20.34	20.46	20.13	20.2	20.31	20.37	20.42	20.44	20.96	20.09	20.16	20.28	20.36	20.39
mtDNA	25.23	25.31	25.35	25.42	25.45	25.55	24.9	24.92	25.66	25.67	25.52	25.64	26.33	25.58	25.61	25.94	26.48	25.89
△ct	5.2	5.22	5.12	5.14	5.11	5.09	4.77	4.72	5.35	5.3	5.1	5.2	5.37	5.49	5.45	5.66	6.12	5.5
△△ct	0.053333	0.073333	-0.026667	-0.006667	-0.036667	-0.056667	-0.376667	-0.426667	0.203333	0.153333	-0.046667	0.053333	0.223333	0.343333	0.303333	0.513333	0.973333	0.353333
2ˆ-△△ct	0.963707	0.950439	1.018656	1.004632	1.025741	1.040060	1.298339	1.344124	0.868541	0.899171	1.032876	0.963707	0.856584	0.788218	0.810378	0.700602	0.509328	0.782773
Relative level	0.963188	0.949927	1.018107	1.004090	1.025188	1.039499	1.297639	1.343400	0.868073	0.898686	1.032319	0.963188	0.856122	0.787793	0.809941	0.700224	0.509053	0.782352

Source Data for Fig. S4G

	c1	c2	c3	c4	c5	c6	h1	h2	h3	h4	h5	h6	n1	n2	n3	n4	n5	n6
nDNA	17.16	16.46	18.06	15.7	16.46	16.83	17.28	18.93	16	17.57	17.96	15.23	18.04	15.85	15.33	19.74	19.83	19.04
mtDNA	24.37	23.35	24.91	22.98	23.23	23.9	24.63	26.3	23.56	24.85	25.13	22.71	25.35	22.9	22.02	26.7	26.56	25.77
△ct	7.21	6.89	6.85	7.28	6.77	7.07	7.35	7.37	7.56	7.28	7.17	7.48	7.31	7.05	6.69	6.95	6.73	6.73
△△ct	0.198333	-0.121667	-0.161667	0.268333	-0.241667	0.058333	0.338333	0.358333	0.548333	0.268333	0.158333	0.468333	0.298333	0.038333	-0.321667	-0.061667	-0.281667	-0.281667
2ˆ-△△ct	0.871557	1.087991	1.118579	0.830278	1.182358	0.960373	0.790955	0.780065	0.683810	0.830278	0.896060	0.722799	0.813191	0.973779	1.249774	1.043671	1.215598	1.215598
Relative level	0.864192	1.078797	1.109126	0.823262	1.172366	0.952257	0.784271	0.773473	0.678031	0.823262	0.888487	0.716691	0.806319	0.965550	1.239212	1.034851	1.205326	1.205326

Original data for western blotting

Homocysteine interferes with Ndufa1 leading to mitochondrial dysfunction through repression of the NAD$^+$ /Sirt1 pathway in the rat brain: A possible link between hyperhomocysteinemia and neurodegeneration

Source Data for Figure 2D

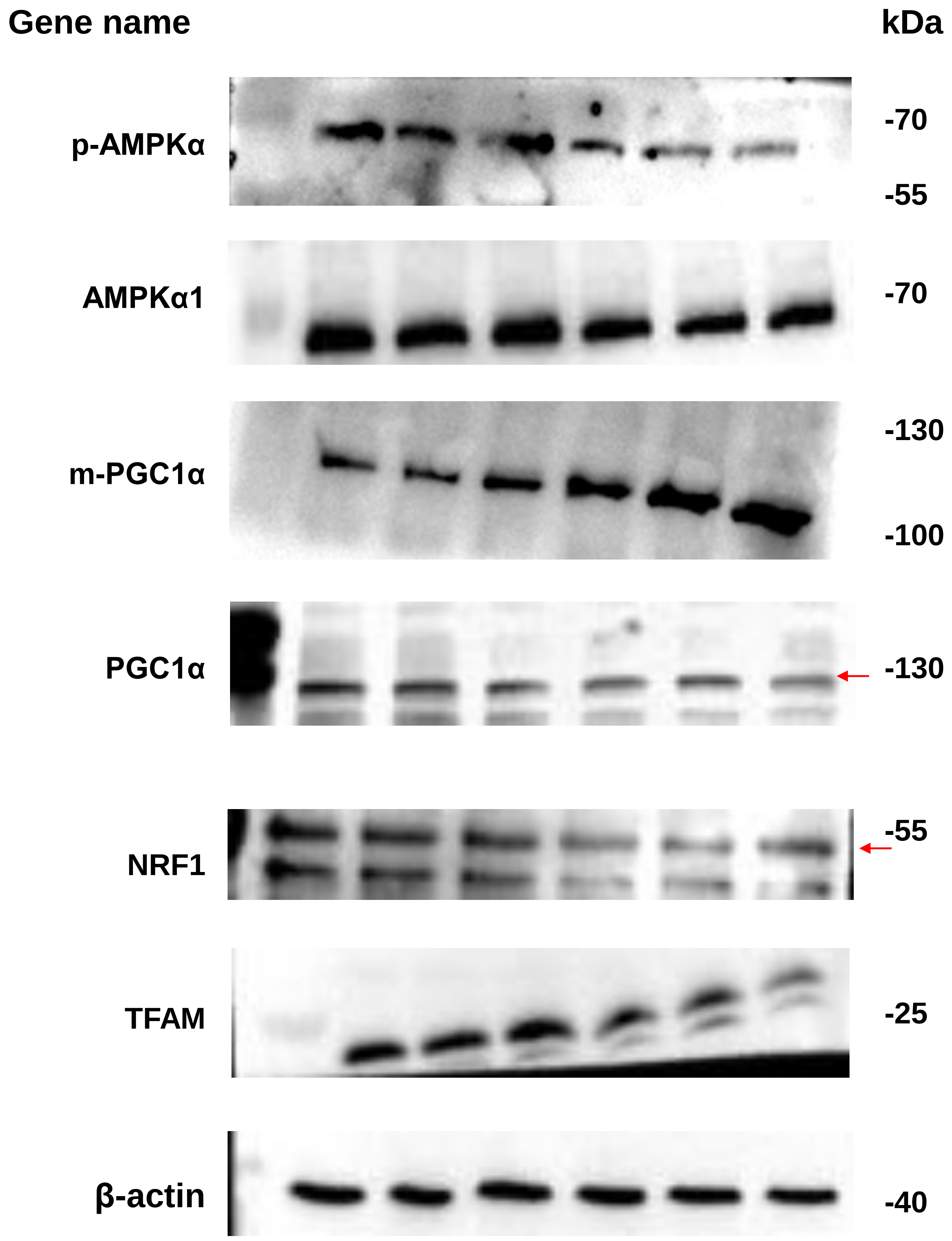

Source Data for Figure 2H

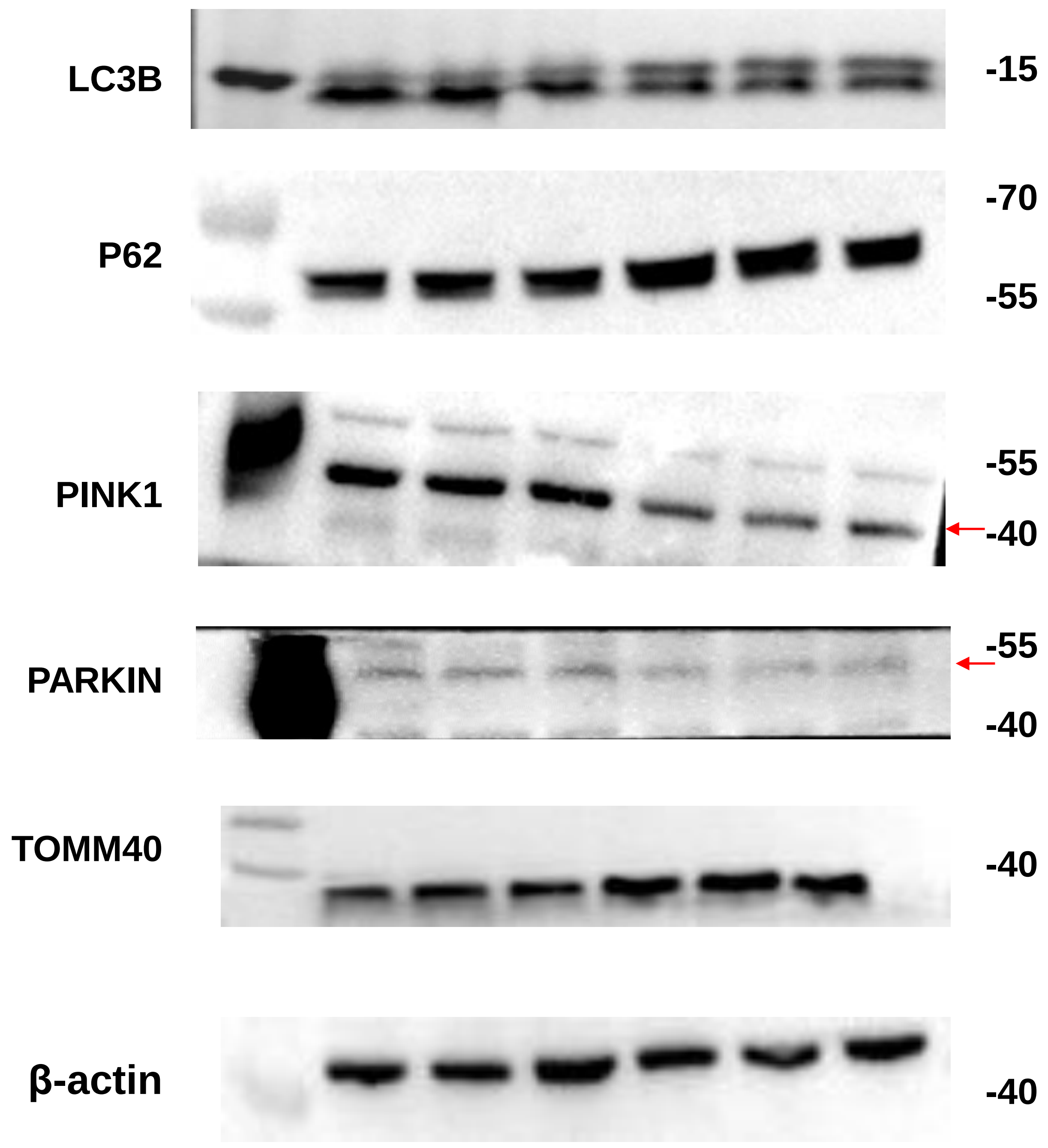

Source Data for Figure 2R

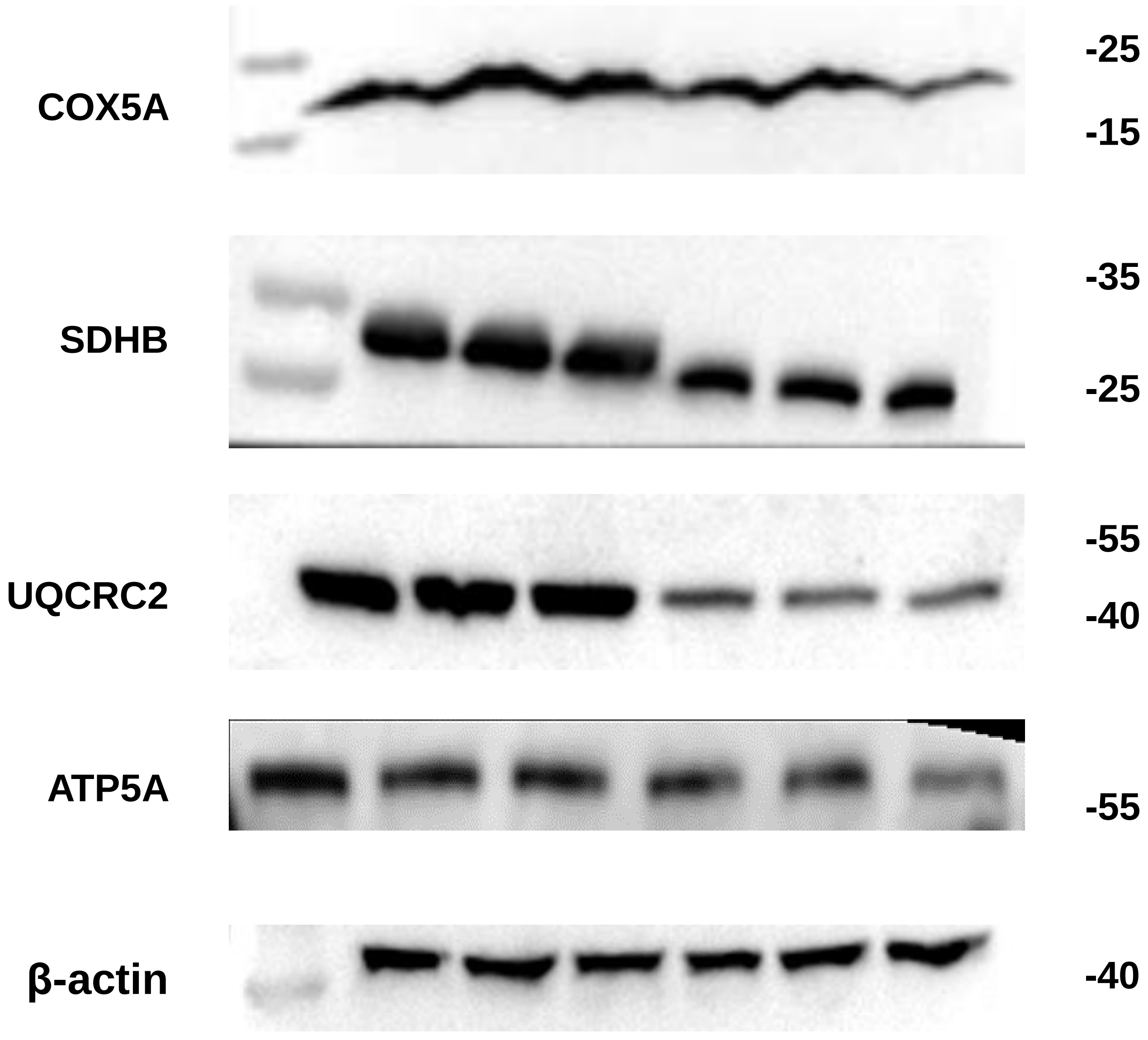

Source Data for Figure 2V

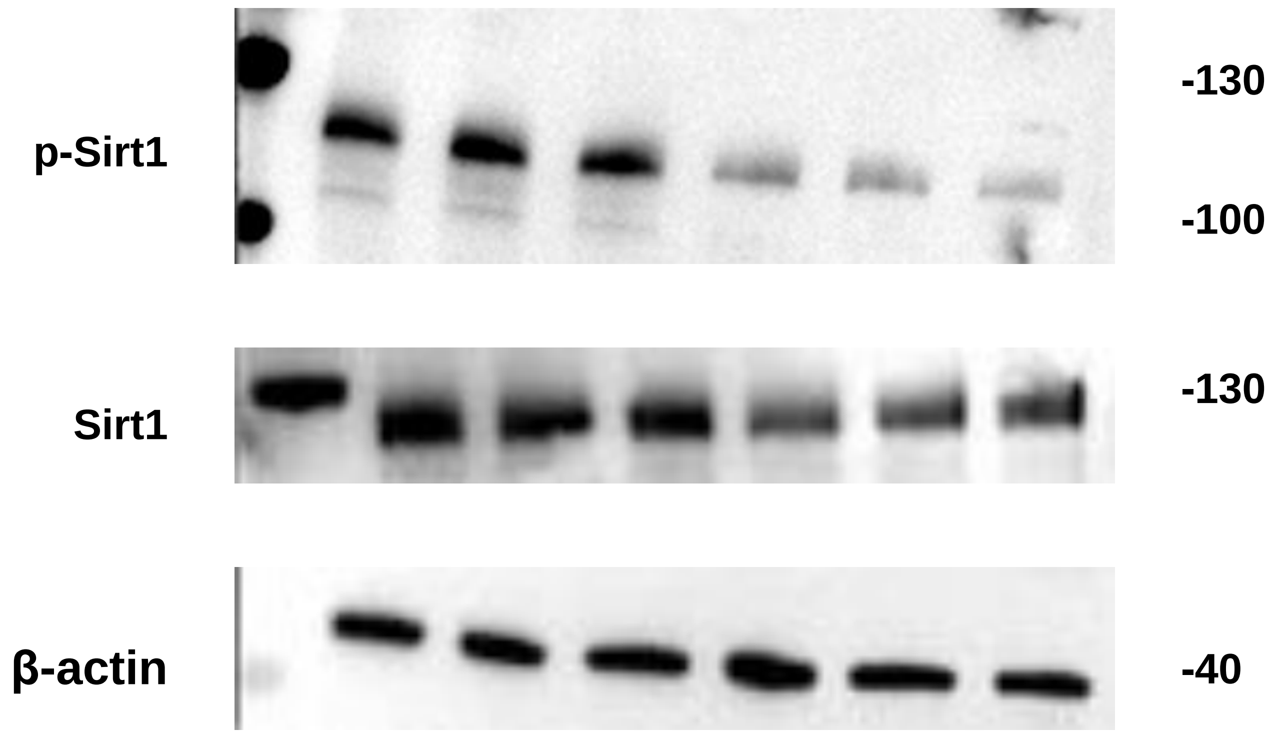

Source Data for Figure 3E

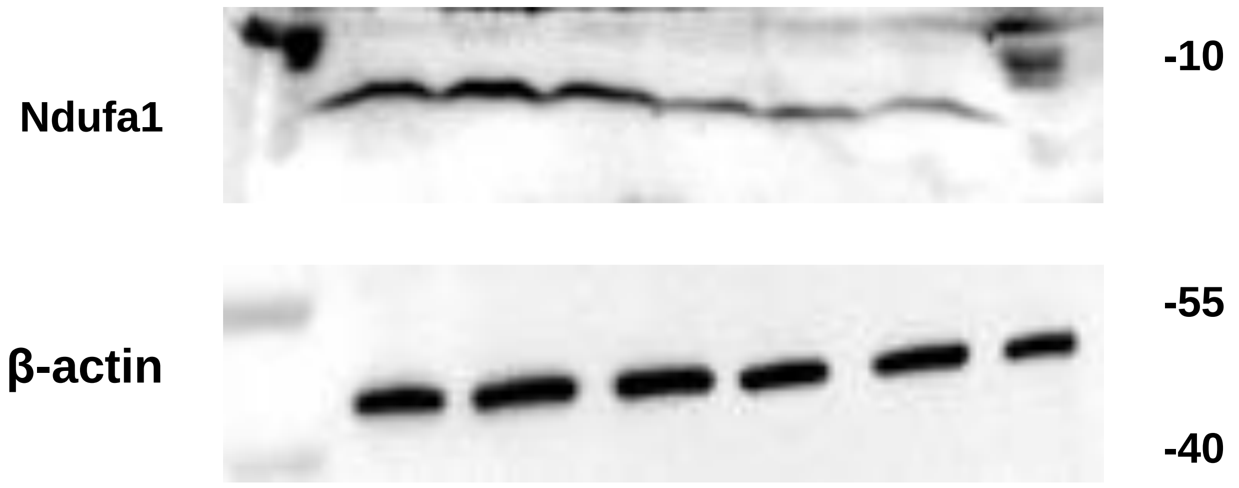

Source Data for Figure 3Q

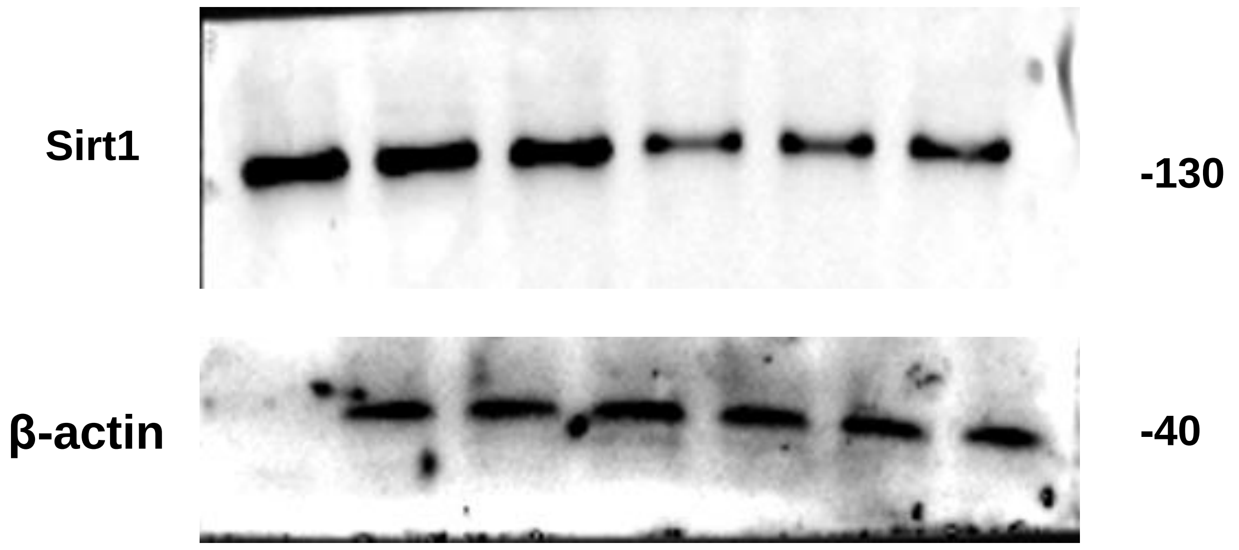

Source Data for Figure 4C

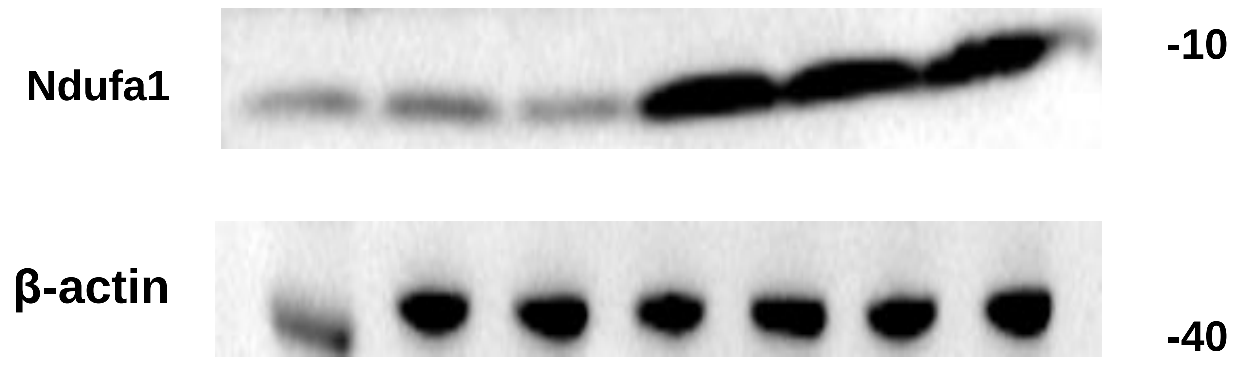

Source Data for Figure 4M

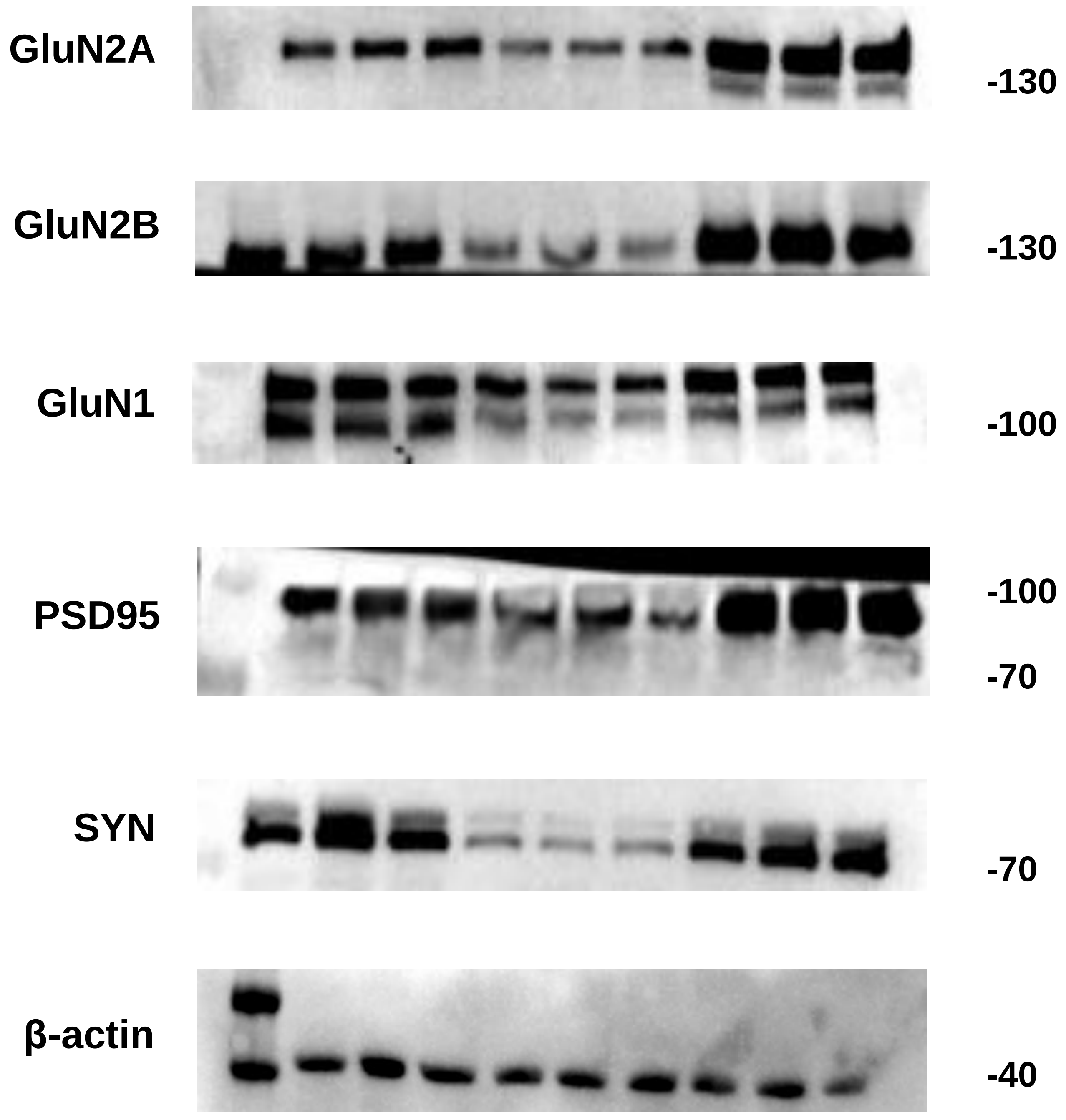

Source Data for Figure 5G

Ndufa1

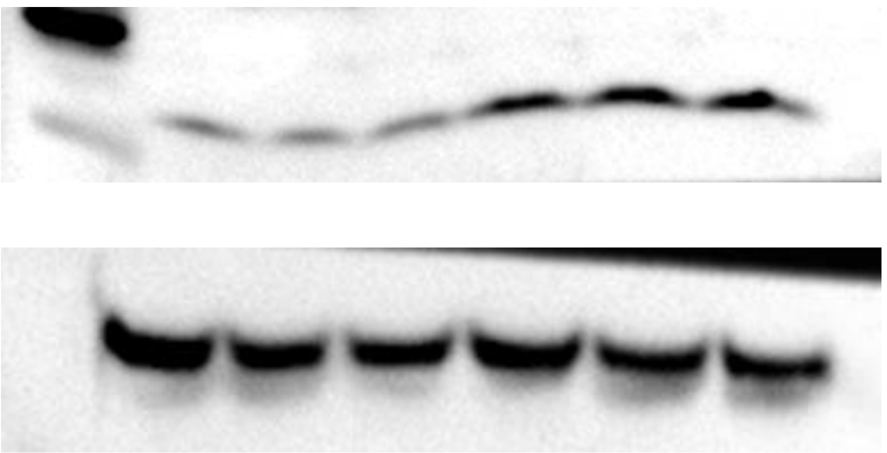

-10

β-actin

-40

Source Data for Figure 6B

Sirt1

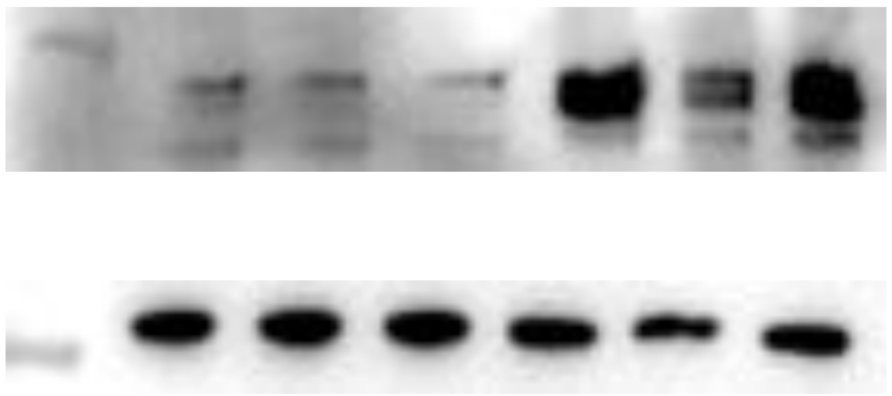

-130

β-actin

-40

Source Data for Figure 6E

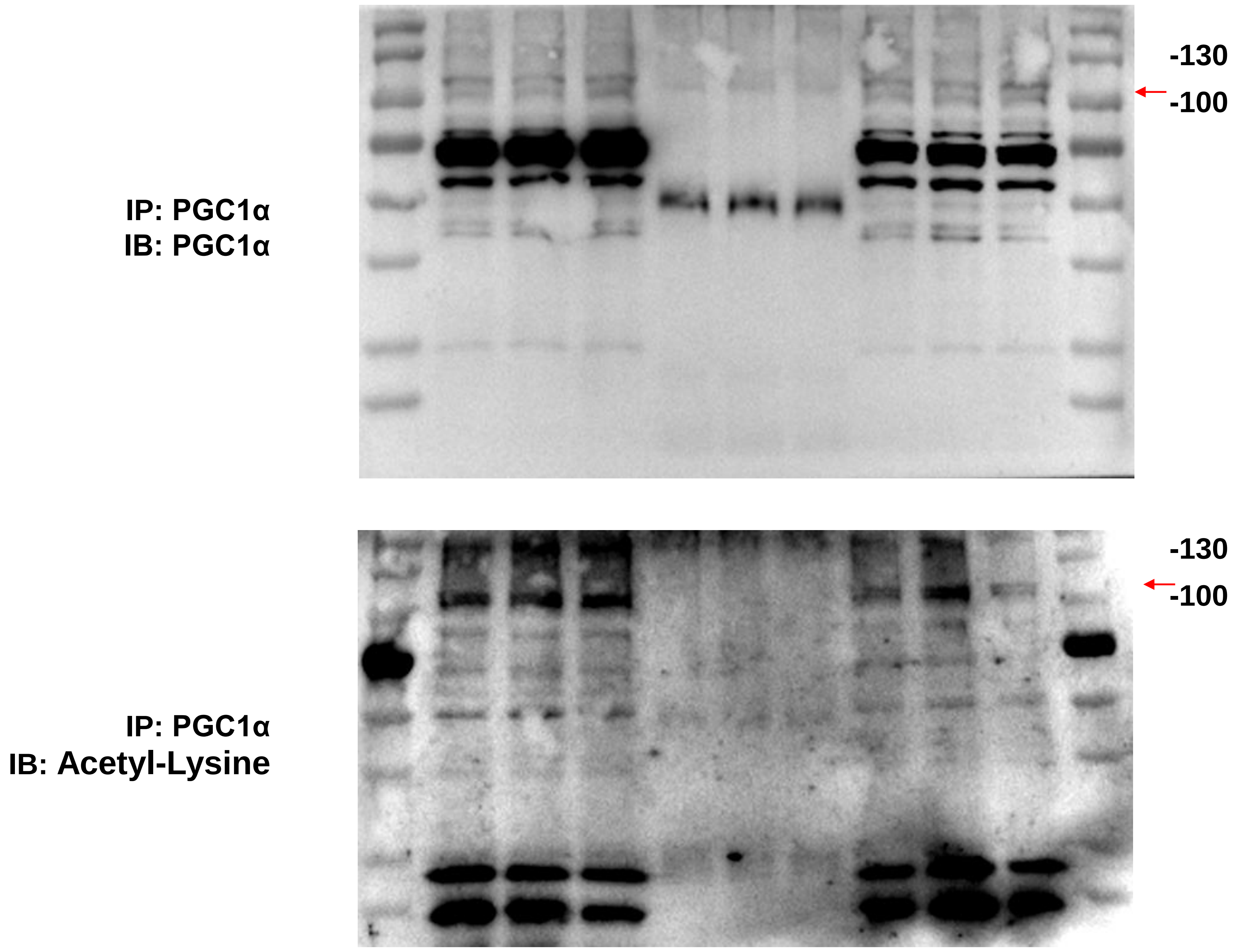

Source Data for Figure 6F

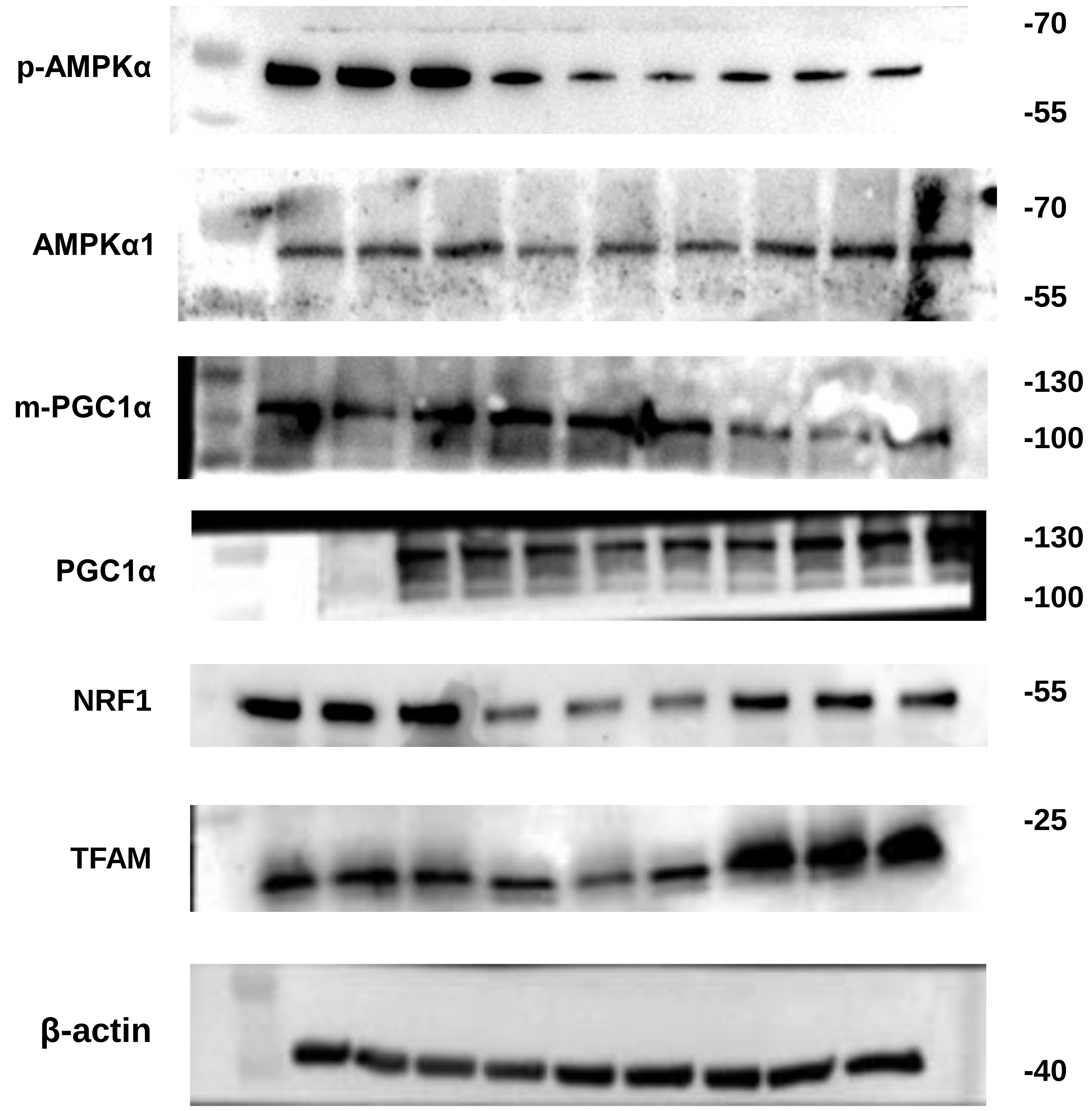

Source Data for Figure 6H

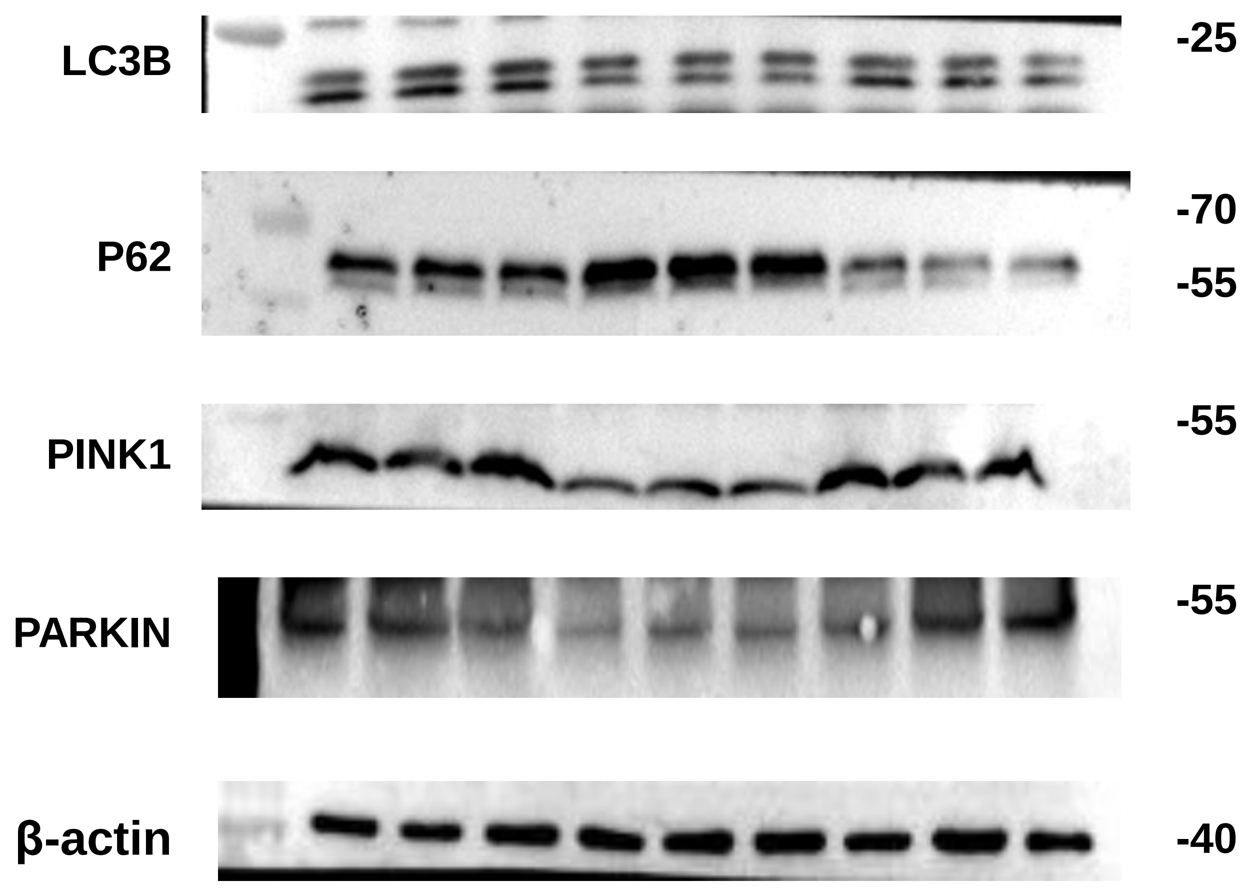

Source Data for Figure 6K

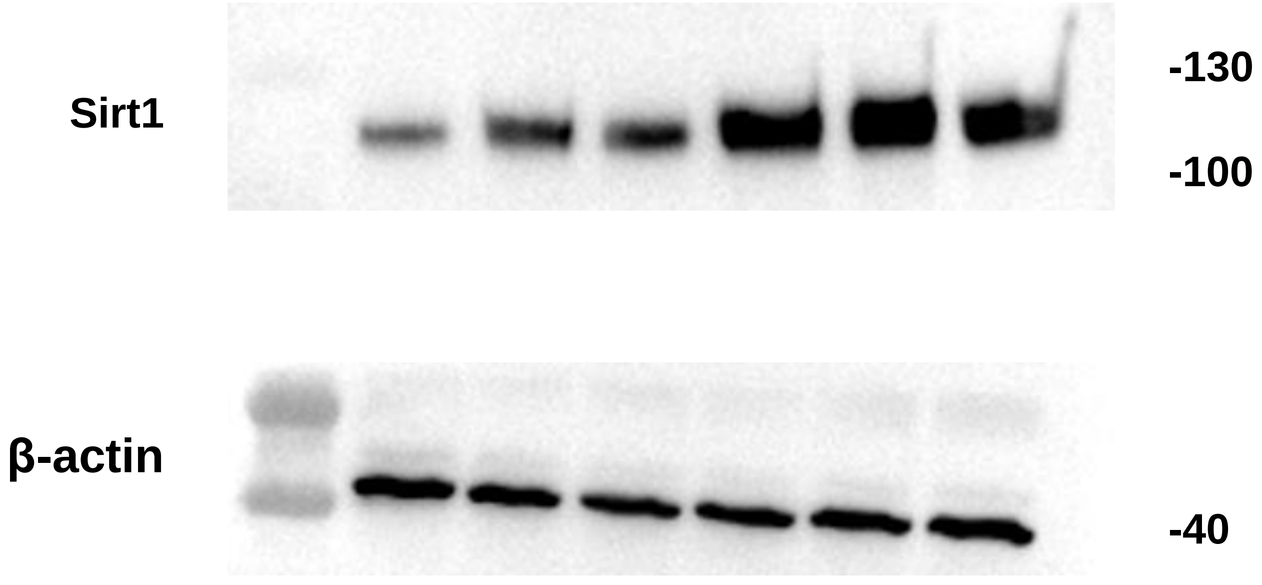

Source Data for Figure 6M

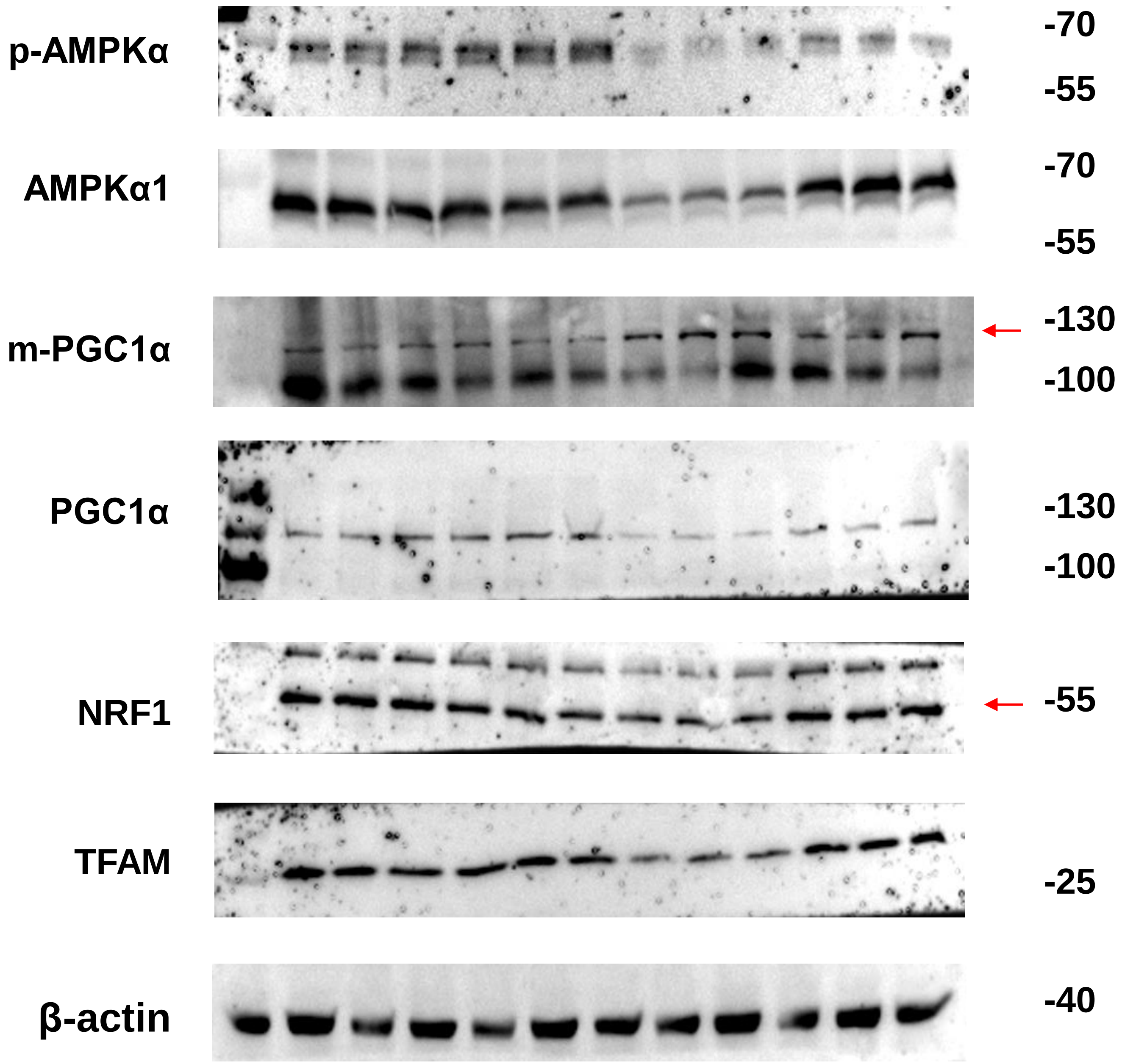

Source Data for Figure 6Q

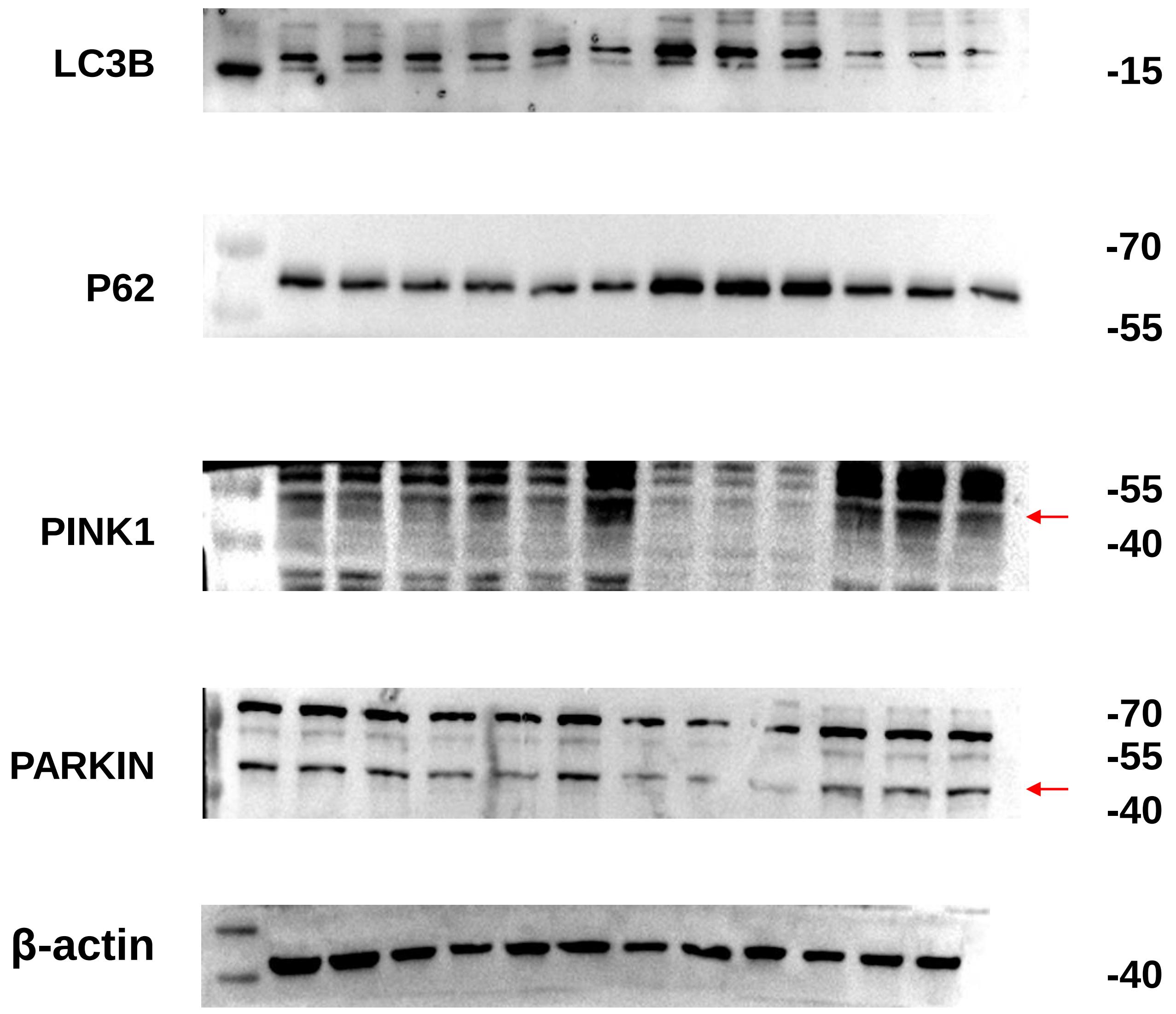

Source Data for Figure 7I

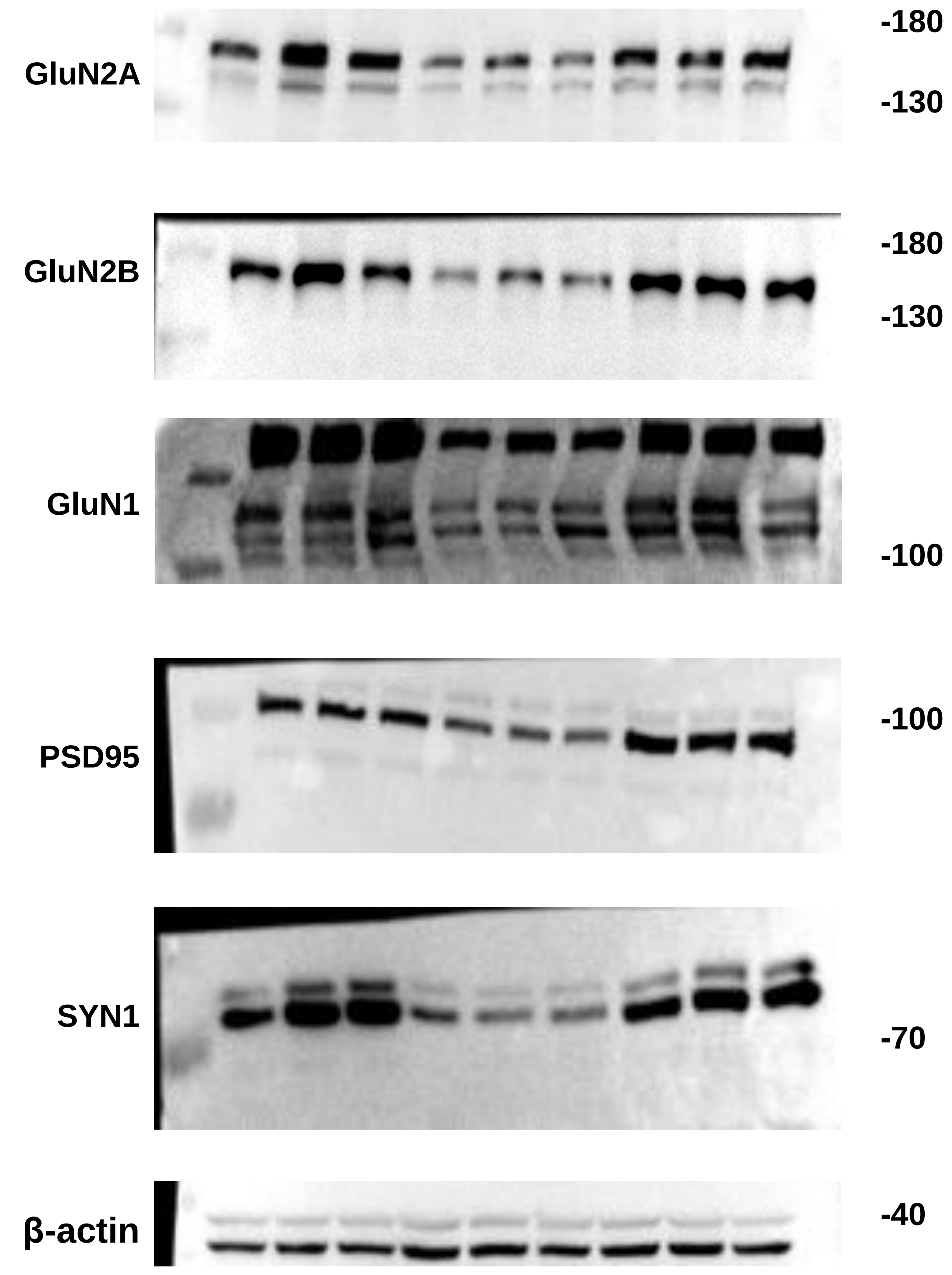

Source Data for Figure 7M

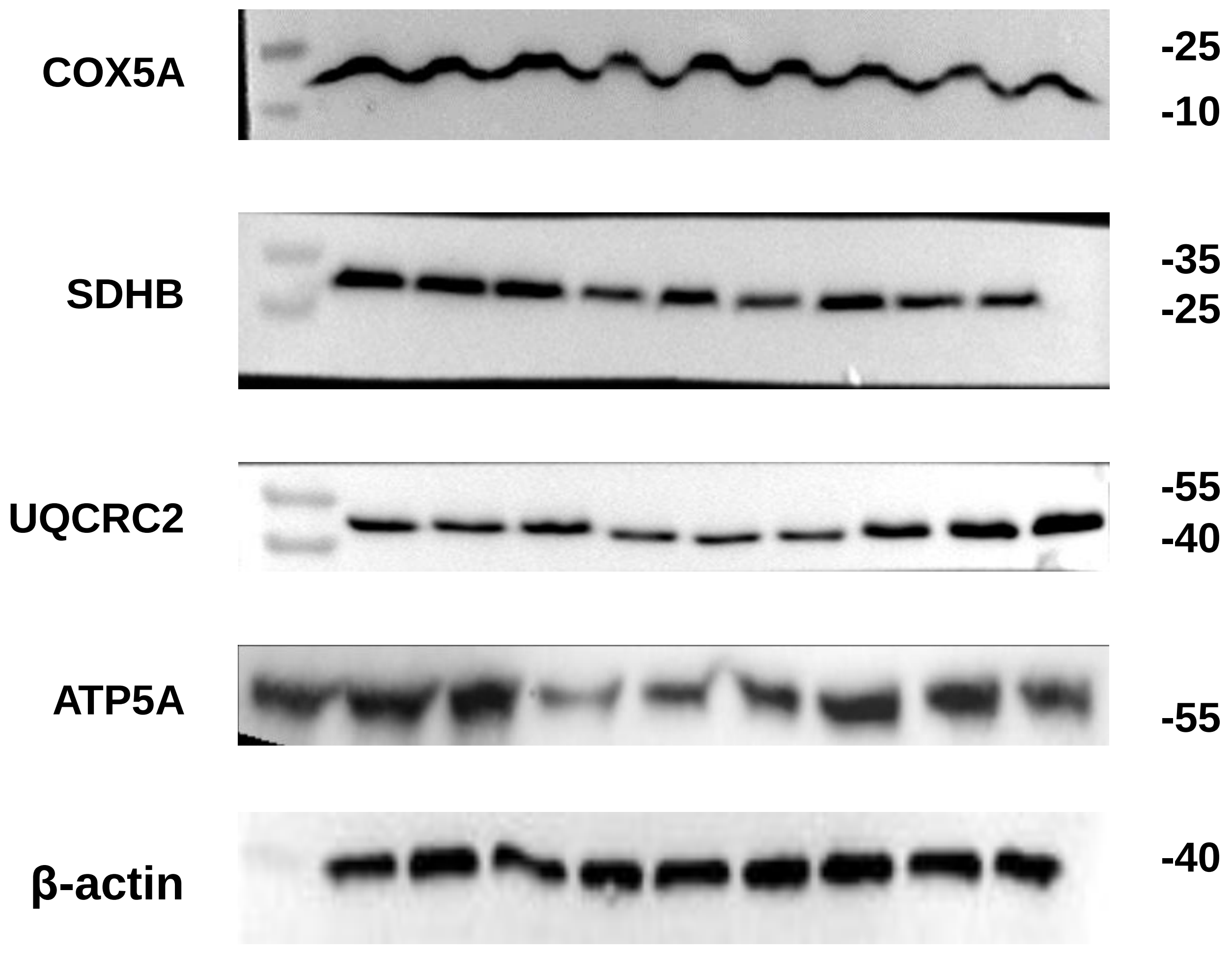

Source Data for Figure 8F

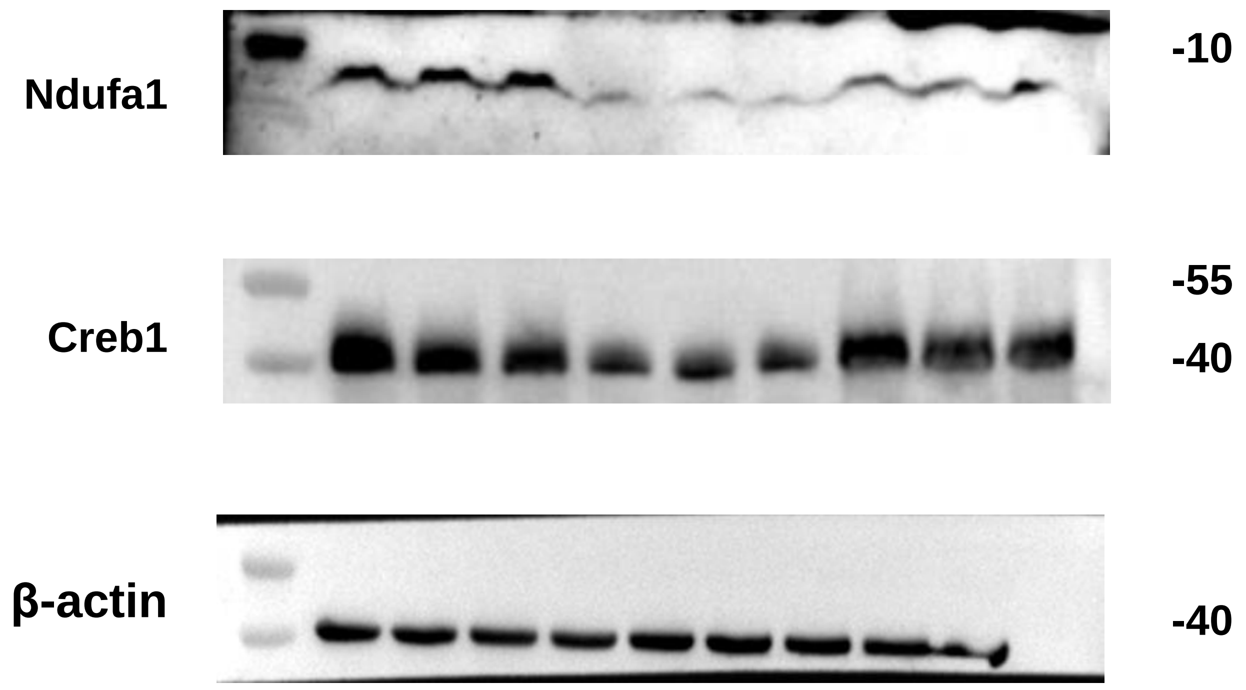

Source Data for Figure 8L

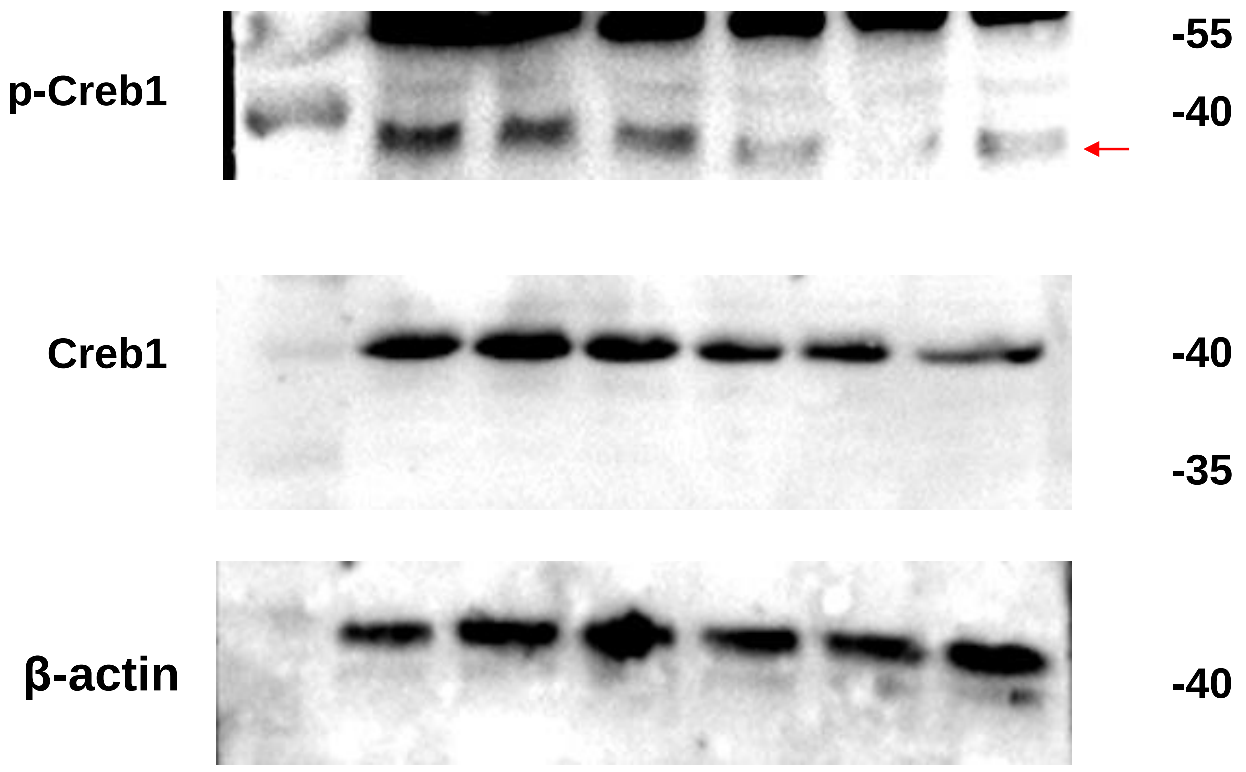

Source Data for Figure 8Q

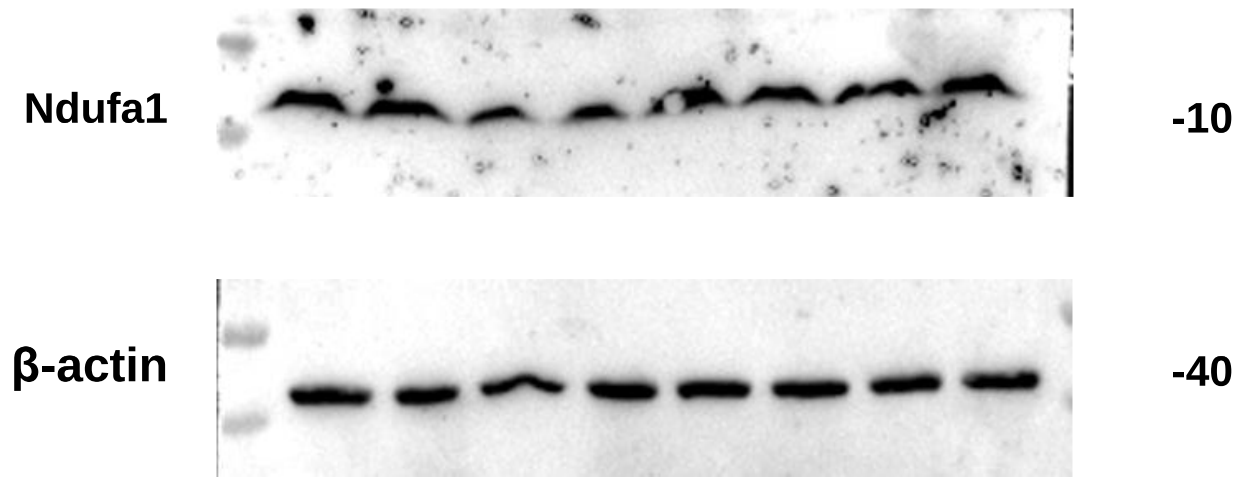

Source Data for Figure S4B

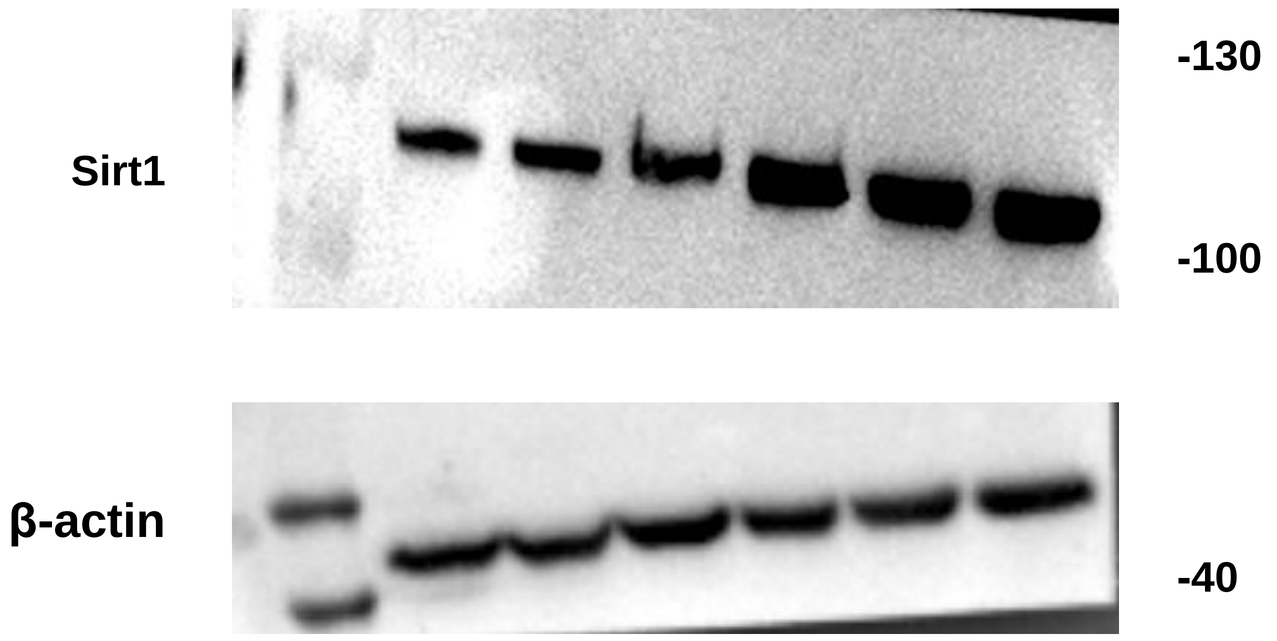

Source Data for Figure S4H

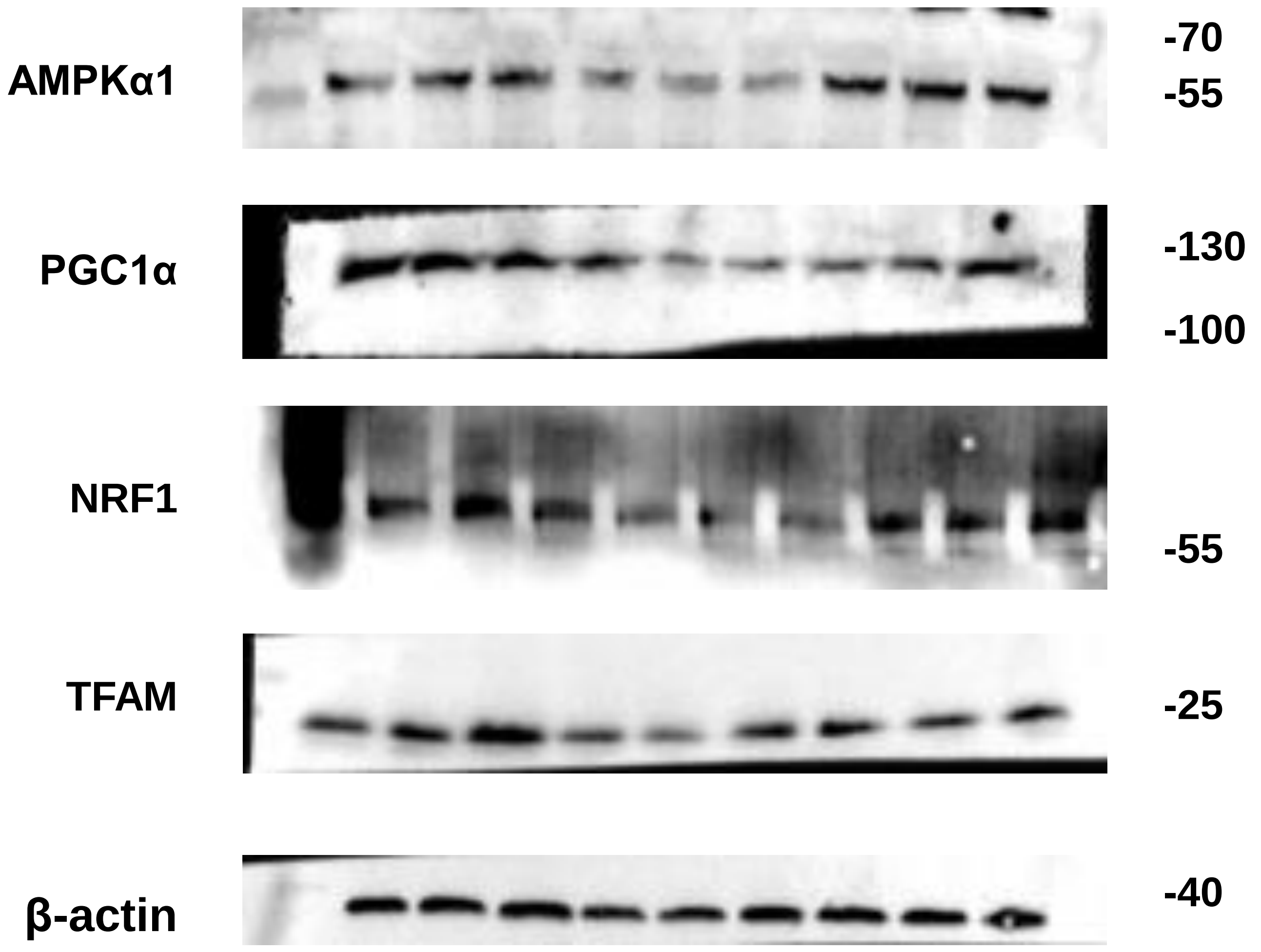

Source Data for Figure S4J

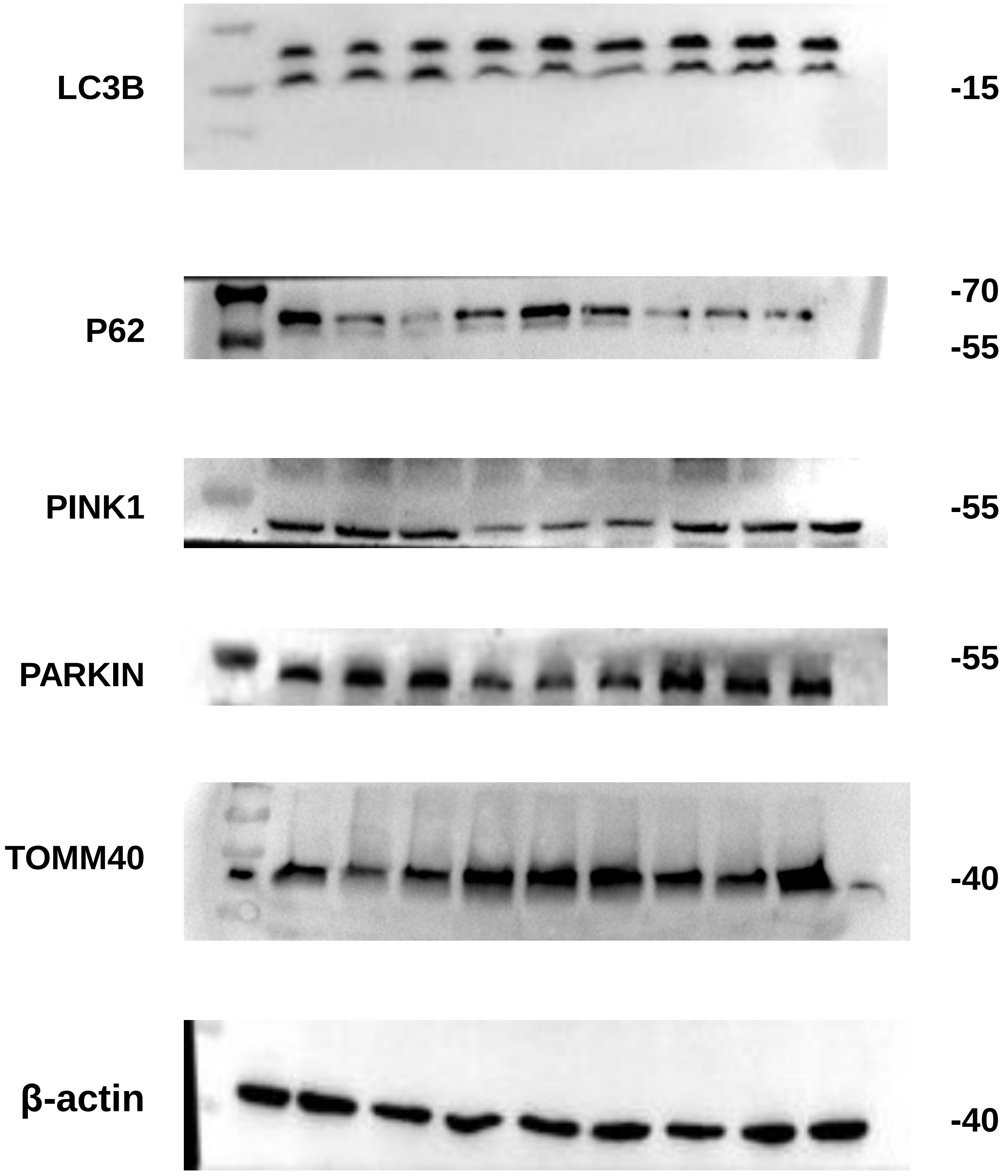

Supplement: Supplementary file 2 — Original data [file 41419_2025_7834_MOESM2_ESM.pdf]
